# Regulatory protein HilD stimulates *Salmonella* Typhimurium invasiveness by promoting smooth swimming via the methyl-accepting chemotaxis protein McpC

**DOI:** 10.1038/s41467-020-20558-6

**Published:** 2021-01-13

**Authors:** Kendal G. Cooper, Audrey Chong, Laszlo Kari, Brendan Jeffrey, Tregei Starr, Craig Martens, Molly McClurg, Victoria R. Posada, Richard C. Laughlin, Canaan Whitfield-Cargile, L. Garry Adams, Laura K. Bryan, Sara V. Little, Mary Krath, Sara D. Lawhon, Olivia Steele-Mortimer

**Affiliations:** 1 Laboratory of Bacteriology, Rocky Mountain Laboratory, National Institutes of Allergy and Infectious Diseases, National Institutes of Health, Hamilton, MT 59840 USA; 2 NIAID Bioinformatics and Computational Biosciences Branch, Rocky Mountain Laboratory, National Institutes of Allergy and Infectious Diseases, National Institutes of Health, Hamilton, MT 59840 USA; 3 NIAID RML Research Technologies Section, Genomics Unit, Rocky Mountain Laboratory, National Institutes of Allergy and Infectious Diseases, National Institutes of Health, Hamilton, MT 59840 USA; 4 Department of Biological and Health Sciences, Texas A&M University-Kingsville, Kingsville, TX 78363 USA; 5 Department of Veterinary Large Animal Clinical Sciences, Texas A&M College of Veterinary Medicine and Biomedical Sciences, College Station, TX 77843 USA; 6 Department of Veterinary Pathobiology, Texas A&M College of Veterinary Medicine and Biomedical Sciences, College Station, TX 77843 USA; 7 Present Address: GlaxoSmithKline, Hamilton, MT 59840 USA

**Keywords:** Bacterial pathogenesis, Cellular microbiology, Pathogens

## Abstract

In the enteric pathogen *Salmonella enterica* serovar Typhimurium, invasion and motility are coordinated by the master regulator HilD, which induces expression of the type III secretion system 1 (T3SS1) and motility genes. Methyl-accepting chemotaxis proteins (MCPs) detect specific ligands and control the direction of the flagellar motor, promoting tumbling and changes in direction (if a repellent is detected) or smooth swimming (in the presence of an attractant). Here, we show that HilD induces smooth swimming by upregulating an uncharacterized MCP (McpC), and this is important for invasion of epithelial cells. Remarkably, in vitro assays show that McpC can suppress tumbling and increase smooth swimming in the absence of exogenous ligands. Expression of *mcpC* is repressed by the universal regulator H-NS, which can be displaced by HilD. Our results highlight the importance of smooth swimming for *Salmonella* Typhimurium invasiveness and indicate that McpC can act via a ligand-independent mechanism when incorporated into the chemotactic receptor array.

## Abstract (web summary)

Protein HilD of *Salmonella* Typhimurium coordinates motility and host cell invasion by upregulating flagellar genes and a secretion system. Here, Cooper et al. show that HilD also modulates swimming behaviour by upregulating a subunit of the chemotactic receptor array, and this is important for invasion of epithelial cells.

## Introduction

Genetically diverse groups of pathogenic microorganisms rely upon directed motility, i.e. chemotaxis, to optimally colonize host tissues. Chemotaxis allows motile bacterial cells to navigate through complex environments, such as the mammalian gastrointestinal tract. In the model organisms *Escherichia coli* and *Salmonella*, bacteria swim in a random pattern produced by alternating counterclockwise (CCW) and clockwise (CW) flagellar rotation. Chemoreceptors detect attractants or repellents and stimulate responses through a signaling cascade that controls the direction of the flagellar motor. Attractant gradients extend the length of time flagellar motors rotate CCW, resulting in more smooth swimming in a favorable direction, while repellents cause an increase of CW rotations, resulting in more tumbling and changes in direction. Chemotaxis is required for *Salmonella enterica* serovar Typhimurium (STm) growth in the lumen of the inflamed gut^1,2^, however, non-chemotactic smooth swimming mutants are more invasive^3–5^ suggesting that repression of chemotaxis could be advantageous under certain conditions.

*Salmonella enterica* serovars, including STm, are gut-adapted Gram-negative bacteria that cause disease in a wide variety of vertebrate species. In humans, STm typically causes a self-limiting diarrhea although it can cause severe systemic disease in immune-compromised individuals. Following ingestion, the bacteria colonize the small intestine, a step that is facilitated by the *Salmonella* pathogenicity island 1 (SPI1) and flagellar motility^6,7^. SPI1 encodes a type III secretion system (T3SS1), required for invasion of intestinal epithelial cells and gut inflammation, as well as several transcriptional regulators^8^. The SPI1 regulon is induced in the gut^9–11^, via mechanisms that are not fully understood, or under certain in vitro growth conditions^12–14^, which has enabled characterization of many of the players and revealed transcriptional cross talk with motility genes^15–24^. The SPI1-encoded transcription factors HilA and HilD are the dominant regulators of SPI1 expression. HilD activates HilA^16,25^ and also directly activates transcription of the flagellar master operon *flhDC* by binding upstream of the P5 promoter^21^. In fact, many flagellar proteins also target HilA or HilD^14,26^. Both SPI1 and flagellar genes exhibit bistable expression, i.e, expressing and non-expressing bacteria coexist in the same populations.

In this work, we examine the role of chemotaxis in STm interactions with the intestinal epithelium. We hypothesize that invasion-primed (SPI1-induced) STm may preferentially navigate towards the gut epithelium. By investigating the HilD regulon and focusing on both the ability to invade epithelial cells and motility, we identify the methyl-accepting chemotaxis protein (MCP), McpC, as a HilD-regulated protein that induces smooth swimming in T3SS1-expressing bacteria thus enhancing their net movement toward the mucosal epithelium. Our findings reveal a mechanism of directed motility that optimizes colonization of the gastrointestinal tract by *Salmonella*.

## Results

### In SPI1-induced STm, HilD increases internalization into host cells

**Fig. 1:**
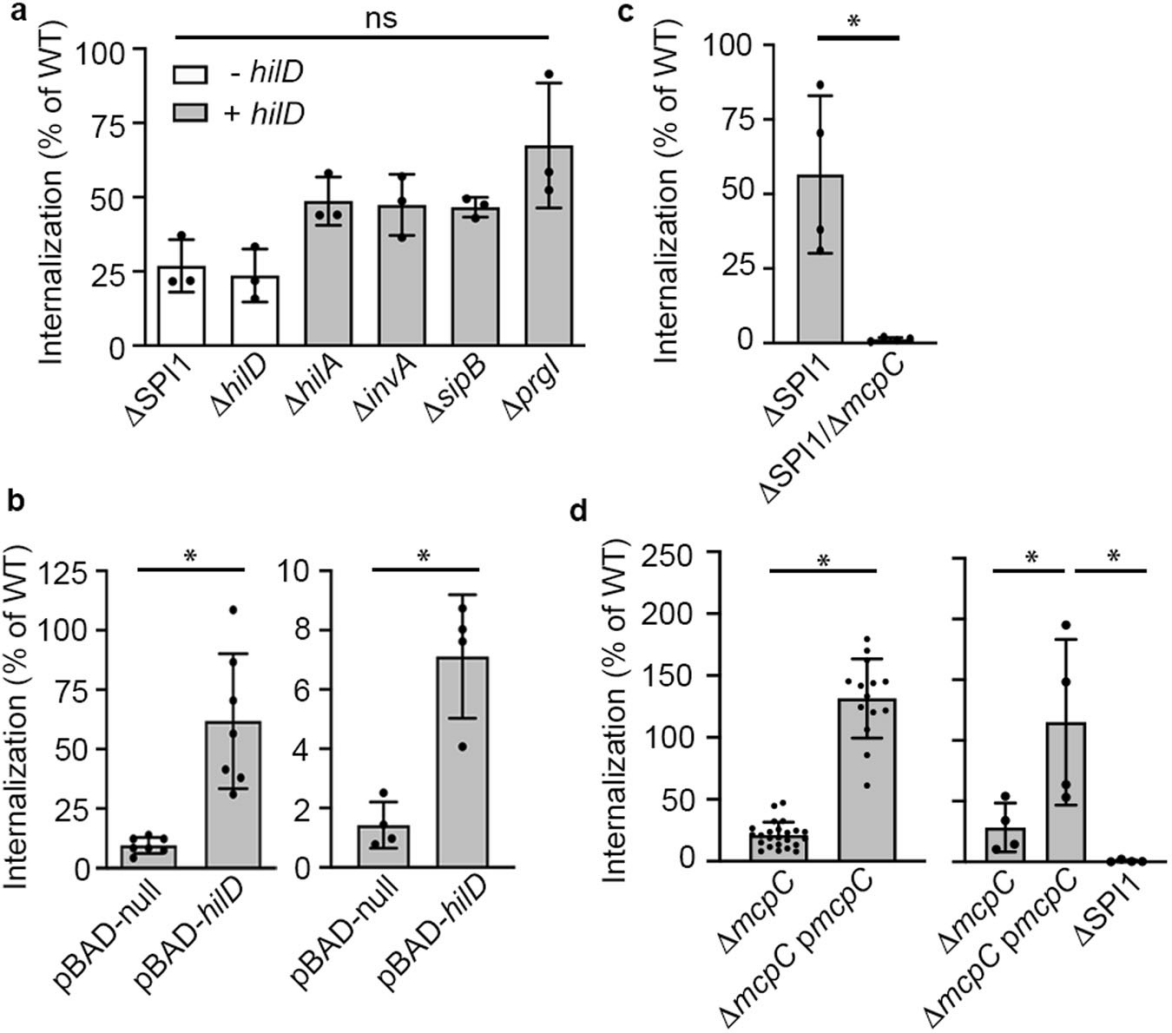
HilD has contributions to invasion efficiency dependent on *mcpC*. **a** Internalization (defined as the % inoculum) at 2 h post-infection (pi) in human monocyte-derived macrophages (HuMDM). *n* = 3, independent experiments with the mean ± SD shown. + or – *hilD* indicates the presence or absence of *hilD*, respectively. **b** Internalization data of HuMDM (2 h pi, left) or C2Bbe1 (1.5 h pi, right) infected with ΔSPI1 induced for null or *hilD* expression. The mean ± SD are shown from *n* = 7 independent donors (HuMDM) or *n* = 4 independent experiments (C2Bbe1). Statistical significances were determined using one-way Anova followed by Tukey’s multiple comparisons. In HuMDM, ΔSPI1 pBAD-null vs. ΔSPI1 pBAD-*hilD*: *p* = 0.005, in C2Bbe1, ΔSPI1 pBAD-null vs. ΔSPI1 pBAD-*hilD*: *p* = 0.02. **c** Internalization at 2 h pi of HuMDM comparing the ΔSPI1 mutant vs. ΔSPI1/Δ*mcpC*, each containing pBAD-*hilD* and induced for *hilD* expression. The mean ± SD of *n* = 4 independent experiments is shown. Statistical significance was determined using one-way Anova followed by Tukey’s multiple comparisons (ΔSPI1 pBAD-*hilD* vs. ΔSPI1/Δ*mpcC* pBAD-*hilD*: *p* = 0.045). **d** Internalization at 1.5 h pi into HeLa cells (left) or C2Bbe1 (right) of the indicated strains. The mean ± SD in HeLa cells are shown from *n* = 24 independent experiments comparing WT and Δ*mpcC* and *n* = 14 of these included Δ*mpcC* p*mpcC*. Statistical significances were determined by one-way Anova with a mixed effects model if a value was missing, followed by Tukey’s multiple comparisons (WT vs. Δ*mpcC*: *p* = 0.0001, Δ*mpcC* vs. Δ*mpcC* p*mpcC*: *p* = 0.0001, WT vs. Δ*mpcC* p*mpcC*: *p* = 0.003). The mean ± SD in C2Bbe1 cells are shown from *n* = 4 independent experiments. Statistical significances were determined by one-way Anova followed by Tukey’s multiple comparisons (Δ*mpcC* vs. Δ*mpcC* p*mpcC*: *p* = 0.02, ΔSPI1 vs. Δ*mpcC* p*mpcC*: *p* = 0.004). Source data are provided as a Source Data file. Growth curves for these strains are included in the Supplementary Information. ns = not significant, *p < 0.05.

To determine if the HilD regulon can control motility towards host cells in SPI1-induced bacteria, we performed internalization experiments using macrophages, which can take up *Salmonella* via both T3SS1-dependent and T3SS1-independent mechanisms. Since the contribution of chemotaxis and/or motility in invasion can be subtle, we compared a *hilD* deletion mutant (Δ*hilD*) to a strain lacking the entire SPI1 (ΔSPI1), which also lacks *hilD*, and the wild-type (WT) strain. As expected, since HilD is required for T3SS1 expression, mutants were internalized into human monocyte-derived macrophages (HuMDM) at a much lower level than WT (Fig. 1a). We next looked at a series of mutants that express *hilD* but are defective in T3SS1 expression (Δ*hilA*) or function (Δ*invA*, Δ*sipB*, and Δ*prgI*). All of these mutants were internalized slightly better than the ΔSPI1 and Δ*hilD* strains, although without statistical significance. Nevertheless, episomal expression of *hilD* in the ΔSPI1 strain under the control of an arabinose inducible promoter (ΔSPI1 pBAD-*hilD*) resulted in increased internalization compared to a control strain (ΔSPI1 pBAD-null) (Fig. 1b, left). A similar effect was seen in C2Bbe1 epithelial cells, into which low levels of T3SS1-independent uptake also occurs (Fig. 1b, right). Thus, in SPI1-induced STm, expression of *hilD* increases both T3SS1-dependent and T3SS1-independent internalization of STm into macrophages and epithelial cells.

### RNAseq reveals that mcpC is a target of HilD

We used RNA sequencing to compare the motility transcriptome in a HilD overexpressing strain compared to WT under SPI1-inducing conditions. HilD^+^ (*hilD*Δ3′UTR) was constructed by removing the *hilD* 3′-untranslated region^27^. As expected, expression of T3SS1 genes and other previously identified HilD targets were upregulated in HilD^+^^28,29^ (Table 1). RNAseq results can be found in Supplementary Data 1. Several motility genes were elevated in HilD^+^, however, only *mcpC*, which encodes for an MCP present in all *Salmonella*, but not in *E. coli*^30^, and previously identified as a potential member of the HilD regulon^28,29^ was significantly upregulated (Table 1).

**Table 1:** RNA sequencing results comparing WT and *hilD*Δ3′UTR.

SL1344 Locus ID	Gene	Pathway	Log2 foldchange	*p*-value	padj
SL1344_RS05360	*sopB*	T3SS1	1.26	1E−13	3E−12
SL1344_RS14880	*prgH*	T3SS1	0.83	2E−05	2E−04
SL1344_RS14890	*hilA*	T3SS1	0.59	6E−04	0.003
SL1344_RS15005	*invF*	T3SS1	0.53	0.006	0.020
SL1344_RS06600	*lpxR*	T3SS1	1.22	4E−08	6E−07
SL1344_RS06605	hypoth.	Lipid A deacylase	1.45	0.008	0.024
SL1344_RS06610	hypoth.	Unknown	1.33	9E−09	1E−07
SL1344_RS22130	hypoth.	Endonuclease	1.39	4E−07	5E−06
SL1344_RS22135	hypoth.	Unknown	0.82	0.018	0.048
SL1344_RS22140	hypoth.	Bacteriophage prot	0.69	0.016	0.043
SL1344_RS16625	*mcpC*	Unknown	0.65	0.003	0.011
SL1344_RS09580	*cheZ*	Motility	0.11	0.325	0.466
SL1344_RS09585	*cheY*	Motility	0.13	0.327	0.467
SL1344_RS09590	*cheB*	Motility	0.15	0.157	0.270
SL1344_RS09615	*motB*	Motility	0.03	0.810	0.881
SL1344_RS09625	*flhC*	Motility	0.29	0.149	0.259
SL1344_RS09630	*flhD*	Motility	0.26	0.210	0.337
SL1344_RS09855	*fliG*	Motility	0.03	0.902	0.945
SL1344_RS09900	*fliP*	Motility	0.11	0.633	0.757
SL1344_RS09910	*fliR*	Motility	0.20	0.527	0.667
SL1344_RS16230	*mcpA*	Motility	0.19	0.343	0.486

Shown are selected genes of the T3SS1, previously identified HilD targets and all upregulated motility genes. *n* = 3 independent samples were collected from each strain. A positive log fold change indicates expression is up in *hilD*Δ3′UTR compared to WT. Significant gene expression differences were identified using DESeq2; *p* values shown were calculated using the Wald test and the *p* value was adjusted (padj) for multiple testing using the Benjamini and Hochberg procedure. Full RNAseq results are found in Supplementary Data 1.

### The chemoreceptor McpC contributes to invasion

Coregulation of *mcpC* with the T3SS1 suggests a role for this chemoreceptor in motility toward host cells, which could account for the ability of *hilD* to increase internalization of the ΔSPI1 mutant (Fig. 1b). Indeed, episomally expressed *hilD* did not rescue internalization of a ΔSPI1/Δ*mcpC* mutant (Fig. 1c), confirming that *mcpC* is required for T3SS1-independent internalization under these conditions. McpC also plays a role in T3SS1-dependent invasion, since Δ*mcpC* had a pronounced invasion defect in HeLa and C2Bbe1 cells that was rescued by episomal expression of *mcpC* under its native promoter (Fig. 1d). Together these data suggest that *mcpC* contributes to invasion efficiency.

### HilD regulates mcpC by derepression of H-NS

**Fig. 2:**
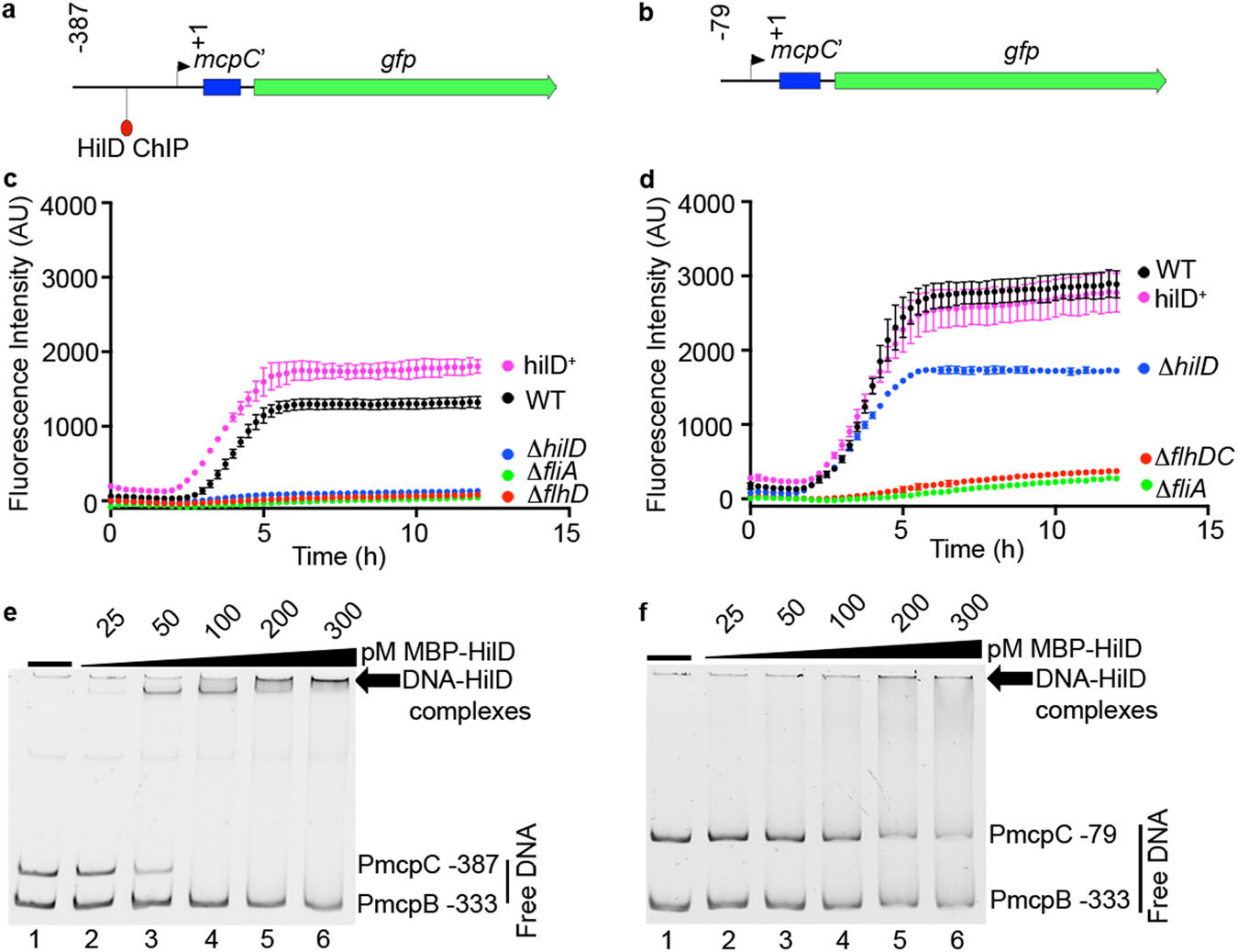
mc*pC* is regulated by a Class III flagellar promoter and dependent on HilD. **a**, **b** Schematic of P*mcpC*-*gfp* transcriptional reporters: **a** P*mcpC* −387 *gfp* and **b** P*mcpC* −79 *gfp*. Numbers indicate the nucleotide locations with reference to the start ATG (+1). *mcpC*’ represents the first 93 nucleotides of coding sequence of *mcpC*. The arrow indicates the predicted Class III promoter. HilD ChIP indicates the peak position of HilD binding identified by ref. ^28^. **c**, **d** GFP fluorescence over time of the indicated strains harboring P*mcpC* −387 *gfp* (**c**) or P*mcpC* −79 *gfp* (**d**). Shown are the mean ± SD of *n* = 3 independent experiments for all strains except Δ*fliA* (*n* = 1). **e**, **f** Electrophoretic mobility shift assay (EMSA) of the indicated promoter regions with purified MBP-HilD. Images are representative *of n* = 3 independent experiments. Source data are provided as a Source Data file.

Flagellar gene expression begins with the master operon *flhDC* that is controlled by a class I promoter. FlhD_4_C_2_ promotes transcription of class II promoters of flagellar assembly genes and the flagellar-specific sigma factor *fliA* (σ28), which directs transcription of class III genes such as flagellin and chemoreceptors^31^. To determine whether *mcpC* is a typical class III flagellar gene, and how HilD contributes to its expression, we monitored *mcpC* promoter activity using a plasmid-based transcriptional GFP reporter. P*mcpC* −387 *gfp* contains the 387 bp intergenic region between the stop codon of the gene *aer* and the translational start of *mcpC* (Fig. 2a). Expression of P*mcpC* −387 *gfp* was assessed and compared in multiple strains (WT, Δ*flhD*, Δ*fliA*, Δ*hilD*, HilD^+^). Strikingly, this assay confirmed the dependence of *mcpC* expression on HilD because GFP fluorescence was virtually undetectable in the Δ*hilD* background and was increased, compared to WT, in the HilD^+^ background. FliA was also required for *mcpC* expression (Fig. 2c). Thus, while *mcpC* like other chemoreceptor genes, is a class III flagellar gene, it is unique in having an additional level of regulatory control that couples its expression to SPI1 induction via HilD.

To further characterize the role of HilD in the regulation of *mcpC*, we constructed a 5′ truncated reporter (P*mcpC* −79 *gfp*) that lacks the predicted HilD-binding site (Fig. 2b). Reporter expression was independent of HilD but still dependent on FliA (Fig. 2d). Levels of HilD-independent reporter expression were lower than that in the WT strain probably due to the loss of HilD-positive effects on flagellar gene expression including *fliA*, through interactions with the *flhDC* P5 promoter^21^. In addition, *mcpC* reporter expression in the *hilD*^+^ strain was increased only in the presence of the HilD-binding site (compare WT and HilD^+^ strains in Fig. 2c, d). These data indicate that the region between −79 to −387, which contains the HilD-binding site, has a repressing effect on *mcpC* expression and support a derepression model by which HilD binding displaces a repressor of *mcpC* expression, i.e. if the upstream region is removed, then HilD binding is no longer required.

To confirm that HilD directly binds the *mcpC* promoter region, we used an electrophoretic mobility shift assay (EMSA). Purified maltose-binding protein HilD fusion protein (MBP-HilD) bound to the full-length 387 bp *mcpC* promoter (P*mcpC*-387, Fig. 2e) but not to the shortened 79 bp regulatory region (P*mcpC*-79, Fig. 2f) or the promoter region of another chemoreceptor (P*mcpB*-333). Thus, binding is specific to the *mcpC* promoter sequence and requires the region between −79 and −387.

**Fig. 3:**
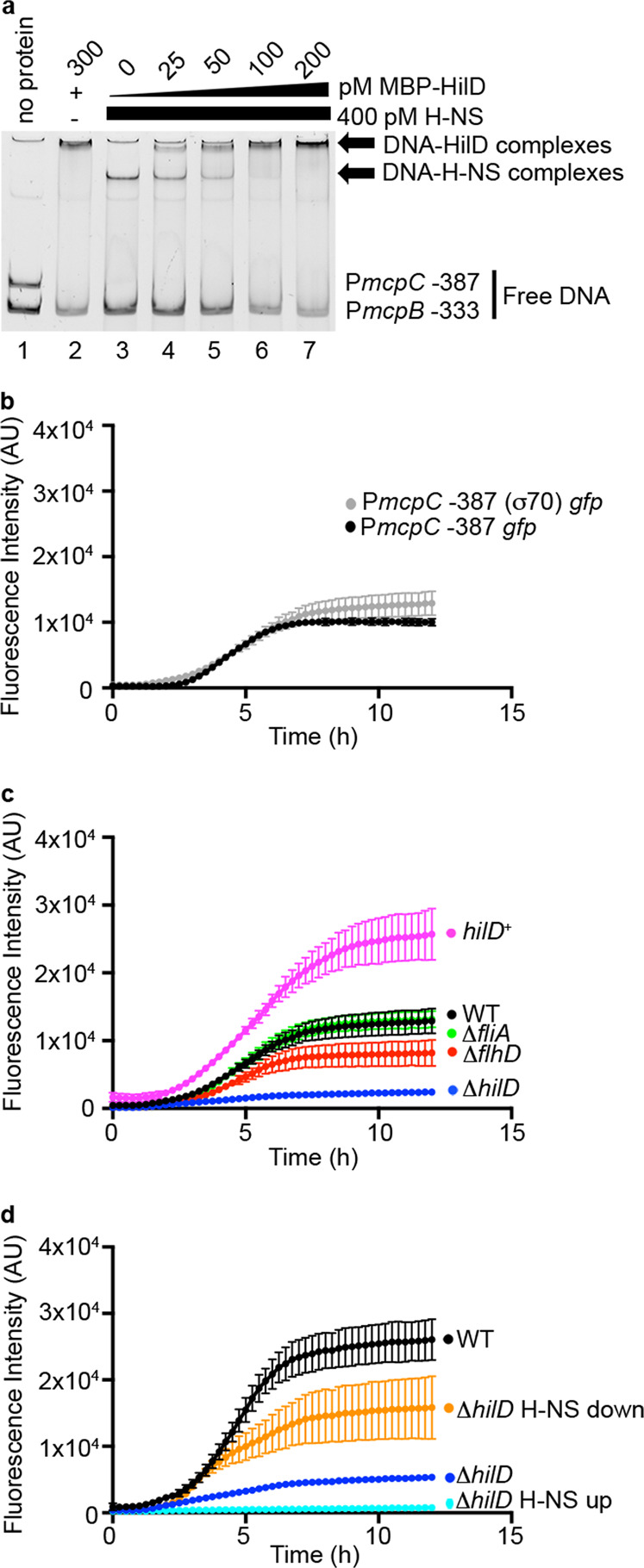
HilD derepresses *mcpC* by displacement of H-NS. **a** Competitive EMSA of the indicated promoter regions with purified MBP-HilD and H-NS. H-NS was added first (lanes 3–7) and increasing amounts of MBP-HilD was subsequently added (lanes 4–7). Similar results were obtained with *n* = 3 independent experiments. **b**–**d** GFP fluorescence over time. **b** WT bacteria containing the indicated reporter. Shown are the mean ± SD of *n* = 3 independent experiments. **c** Indicated strains harboring P*mcpC* (σ70) *gfp*. Shown are the mean ± SD of *n* = 3 independent experiments. **d** Indicated strains harboring P*mcpC* (σ70) *gfp*. H-NS up contains both P*mcpC* (σ70) *gfp* and pMPMT6 *hns*^WT^. H-NS down contains both P*mcpC* (σ70) *gfp* and pMPMT6 *hns*^Q92am^. Source data are provided as a Source Data file.

HilD can displace the global repressor H-NS from DNA^32^ and H-NS-binding sites are located near *mcpC*^33,34^. We found that purified H-NS bound to the region upstream of *mcpC* (P*mcpC-*387) but not to the promoter of *mcpB* (P*mcpB*-333) (Fig. 3a, compare lanes 1 and 3). Furthermore, in a competitive EMSA HilD displaced H-NS from the *mcpC* promoter (lanes 3–7) with full displacement occurring at 50–100 pM (lanes 5 and 6). Thus, HilD and H-NS specifically bind to the regulatory region of *mcpC* and HilD is an antagonist of H-NS binding.

We next analyzed the effects of H-NS in bacteria, using inducible expression of either WT (H-NS^WT^) or dominant negative H-NS (H-NS^Q92am^)^35^ in the presence of P*mcpC* −387 *gfp*. However, since H-NS is required for expression of FliA, the sigma factor required for transcription of *mcpC*^36,37^ (Fig. 2) we altered the reporter to replace the FliA (σ28) recognition site with a σ70 recognition sequence. This FliA-independent (σ70-dependent) *mcpC*-*gfp* reporter (P*mcpC* −387 (σ70)) displayed similar expression kinetics and magnitude to that of the original *mcpC* reporter (Fig. 3b) and no longer required FliA or FlhD (Fig. 3c). Removing FlhD did have a slight detrimental effect on reporter expression, probably due to the loss of the flagellar protein FliZ and its positive impacts on HilD activity^22^. Importantly, *gfp* expression was still dependent on HilD and amplified in the HilD^+^ strain (Fig. 3c). Manipulation of H-NS levels by induction of H-NS^WT^ or H-NS^Q92am^ revealed that increasing H-NS reduced P*mcpC* −387 (σ70) *gfp* expression, while decreasing H-NS resulted in HilD-independent expression (Fig. 3d). These results unequivocally demonstrate that H-NS represses *mcpC* expression and HilD is required to derepress *mcpC* expression under these conditions.

### McpC functions independently of T3SS1 to increase bacteria/host association

**Fig. 4:**
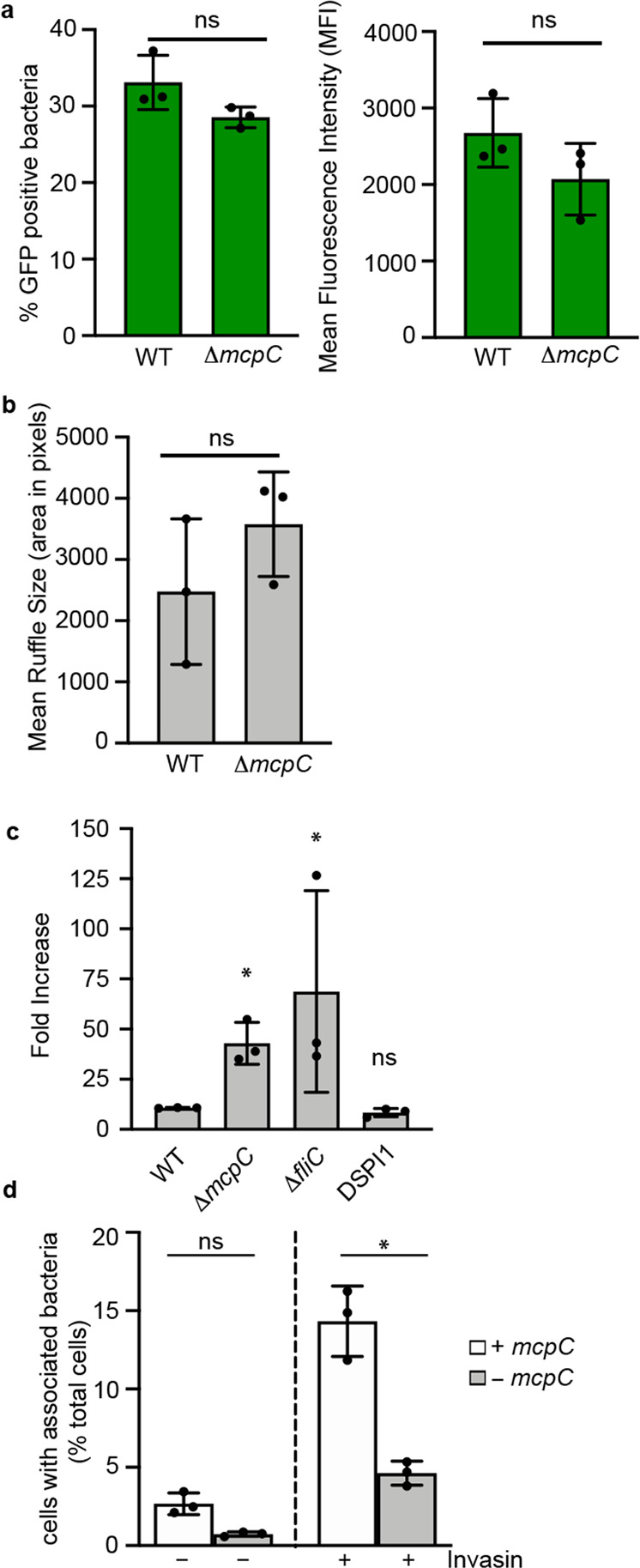
McpC functions independently of T3SS1 to increase bacterial/host cell association. **a** T3SS1 gene expression is not altered in Δ*mcpC*. SPI1-induced bacteria harboring P*prgH gfp* [LVA] were analyzed by flow cytometry (*n* = 3 independent experiments). The mean ± SD percentage of GFP-positive bacteria (left panel) and the mean fluorescence intensity (right panel) are shown. Significance was determined using a two-tailed, paired Student’s *t* test (WT vs. Δ*mcpC*, % GFP positive: *p* = 0.2, WT vs. Δ*mcpC*, MFI: *p* = 0.1). **b** HeLa cells were infected with WT and Δ*mcpC* using centrifugation to synchronize bacterial/host association. Shown are the mean ± SD ruffle sizes from WT infected cells (*n* = 132 cells, examined over three independent experiments) and from Δ*mcpC* infected cells (*n* = 157 cells, examined over three independent experiments). Significance was determined using a two-tailed, paired Student’s *t* test (WT vs. Δ*mcpC*; *p* = 0.4). **c** HeLa cells were infected with the indicated strains with or without centrifugation. Shown are the mean ± SD fold increases in internalization with centrifugation at 1.5 h pi from *n* = 3 independent experiments. Significance was determined by two-way Anova of log-transformed values followed by Tukey’s multiple comparisons (WT vs. Δ*mcpC*; *p* = 0.002, WT vs. Δ*fliC*; *p* = 0.03, WT vs. ΔSPI1: *p* = 0.7). **d** HeLa cells were infected with Δ*invA* or Δ*invA*/Δ*mcpC*, with or without expression of invasin (as indicated) for 6 min. Shown are the mean ± SD percentage of cells with associated bacteria (Δ*invA*; *n* = 1212 cells examined over three independent experiments, Δ*invA* + Invasin; *n* = 1518 cells examined over three independent experiments, Δ*invA*/Δ*mcpC*; *n* = 1173 cells examined over three independent experiments, Δ*invA*/Δ*mcpC* + Invasin; *n* = 1489 cells examined over three independent experiments). Significance was determined by one-way Anova followed by Tukey’s multiple comparisons (Δ*invA* + Invasin vs. Δ*invA*/Δ*mcpC* + Invasin; *p* = 0.0001. Green bars indicate that GFP-positive bacteria were analyzed in the assay. ns not significant, *p < 0.05. Source data are provided as a Source Data file.

We hypothesized that McpC promotes chemotaxis of T3SS1-primed bacteria toward target host cells. To test this, we first ruled out that the invasion defect of Δ*mcpC* is due to aberrant expression or functionality of T3SS1. We evaluated SPI1 gene expression with a *prgH-gfp*[LVA] reporter in WT vs. Δ*mcpC* by flow cytometry and found no differences in the percentage or magnitude of expression (Fig. 4a). Additionally, gentle centrifugation to increase contact of Δ*mcpC* with host cells overcame the invasion defect similar to rescuing a *fliC* mutant and unlike ΔSPI1 (Fig. 4c). Furthermore, the size of T3SS1-induced ruffles in HeLa cells were similar when cells were infected by Δ*mcpC* vs. WT (Fig. 4b), indicating similar magnitude of effector delivery. Altogether, these results indicate that the invasion defect of Δ*mcpC* is due to decreased contact with host cells; once contact is made, normal T3SS1-dependent invasion occurs. To further confirm that McpC is important pre-invasion, we measured the ability of bacteria to make contact with host cells. Heterologous expression of the adhesin invasin from *Yersinia pseudotuberculosis* in the invasion deficient STm strain, Δ*invA*, mediates tight attachment to host cells via interactions between invasin and β1 chain integrin receptors^38^. As expected, invasin expression by STm increased the percentage of cells with associated bacteria over the Δ*invA* mutant alone, indicating that invasin was mediating attachment (Fig. 4d). However, when *mcpC* was deleted from these strains, bacterial attachment was reduced. These results indicate McpC optimizes interaction with host cells.

### McpC is required for optimal colonization in the gastrointestinal tract

**Fig. 5:**
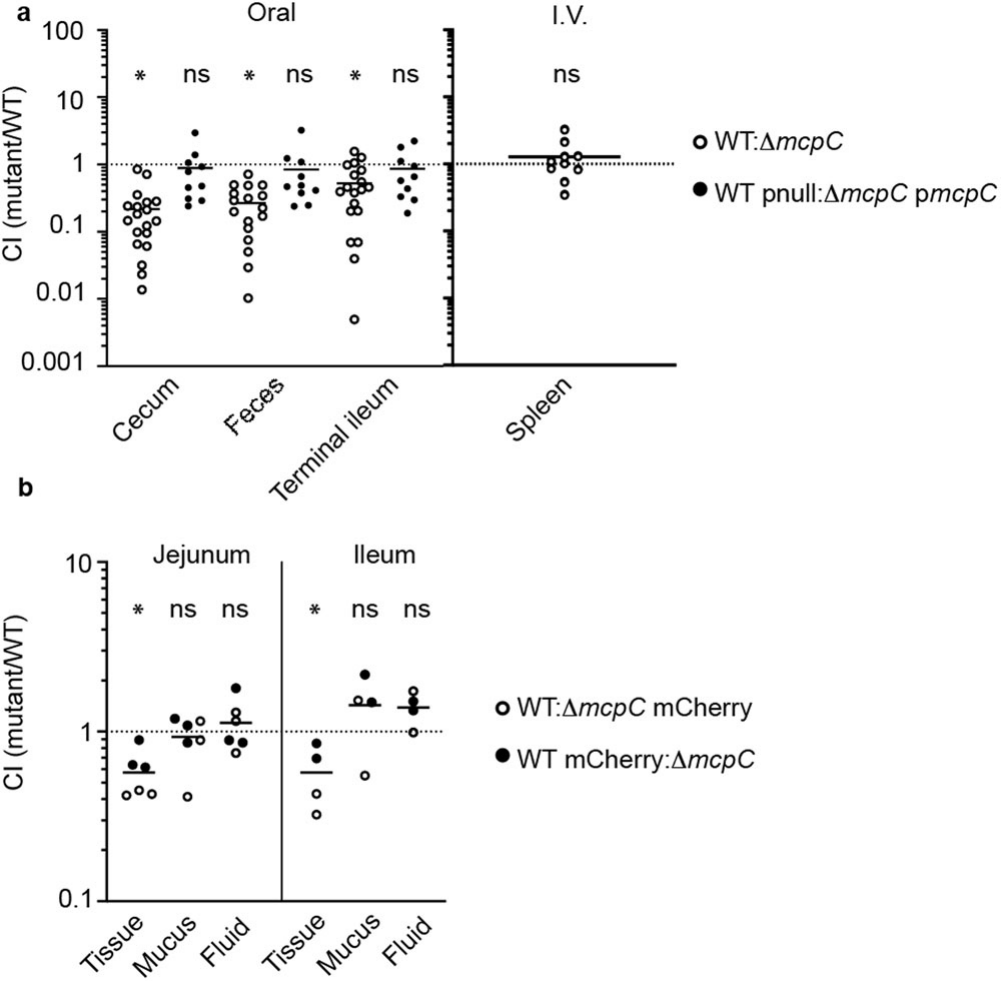
McpC contributes to invasion in the gastrointestinal tract. **a** Streptomycin pretreated C57BL/6 mice were infected orally with a 1:1 mixture of 10^6^ CFU (oral) or 500 CFU (i.v.) each of either WT and Δ*mcpC* or WT carrying an empty plasmid (pnull) and Δ*mcpC* carrying a complementing plasmid (p*mcpC*). For oral infections, the indicated organ CFU loads of either *n* = 10 mice (WT pnull:Δ*mcpC* p*mcpC*) or *n* = 20 mice (WT:Δ*mcpC*), were assessed at 2 d pi. For intravenous infection, the spleen CFU loads of *n* = 10 mice (WT:Δ*mcpC*) were assessed at 4 d pi. Each dot represents the CI from a single mouse with the mean shown. Statistical significances were determined by two-way Anova followed by Sudak’s multiple comparisons on log-transformed CFU values for analyzing multiple tissue types in the gastrointestinal tract (WT vs. Δ*mcpC* in the cecum: *p* = 0.0001, in the feces: *p* = 0.0002, in the terminal ileum: *p* = 0.005; WT pnull:Δ*mcpC* p*mcpC* in the cecum: *p* = 0.5, in the feces: *p* = 0.3, in the terminal ileum: *p* = 0.5). For analyzing statistical significance in the spleen, a two-tailed Wilcoxon test was performed on log-transformed CFU values (WT vs. Δ*mcpC*: *p* = 0.13). **b** Ligated jejunal (*n* = 6 loops from 3 calves) or ileal loops (*n* = 4 loops from 2 calves) were injected with a 1:1 mixture of 10^7^ CFU total of WT and Δ*mcpC*, one of which expressed mCherry as indicated. Each dot represents the CI from an individual loop with the mean shown. Tissue samples (gentamicin-treated biopsies), mucus, or luminal fluid were assessed at 2 h pi. Statistical significance was determined by two-way Anova followed by Sudak’s multiple comparisons on log-transformed CFU values (WT vs. Δ*mcpC* in the jejunal tissue: *p* = 0.007, in the ileal tissue: *p* = 0.02, in the jejunal fluid: 0.9, in the ileal fluid: *p* = 0.9, in the jejunal mucus: *p* = 0.8, in the ileal mucus: *p* = 0.9). ns = not significant, *p < 0.05. Source data are provided as a Source Data file.

To investigate if McpC confers an in vivo advantage, C57BL/6 mice were infected with a 1:1 mixture of wild type and Δ*mcpC*. At 2 days post-infection (pi), the competitive index (CI) showed that Δ*mcpC* was outcompeted by the WT in the cecum, feces, and terminal ileum after oral inoculation of streptomycin pre-treated mice. However, after intravenous inoculation, both strains were recovered in equal amounts 4 days pi in the spleen. Furthermore, an *mcpC* complemented strain was recovered in similar amounts to WT in the gut (Fig. 5a). This indicates that McpC plays an important role in the gastrointestinal tract but not during systemic infection of mice.

The calf ileal loop model^39,40^ has demonstrated a requirement for chemotaxis in the inflamed intestine^1^. Since McpC has a role in early “pre-inflammatory” colonization in the mouse gastrointestinal tract (Fig. 5a), we modified the established calf loop model by using a low inoculum (10^7^ cfu/loop), focusing on an earlier time point (2 h pi) and using a CI approach to maximize sensitivity. For identification by fluorescence microscopy, either WT or Δ*mcpC* expressed mCherry constitutively. In both jejunal and ileal loops, irrespective of which strain expressed mCherry, Δ*mcpC* was recovered in lower amounts than WT in gentamicin-treated tissue but in equal amounts from luminal fluid and mucus (Fig. 5b). Confocal microscopy confirmed that more WT bacteria associated with host cells than the mutant although quantification was not possible due to low numbers of bacteria in the tissues (Supplementary Figs. 1 and 2). These data confirm that, in vivo, *mcpC* is important for invasion of gut epithelium.

### McpC functions to promote CCW flagellar rotational bias in SPI1-induced bacteria

**Fig. 6:**
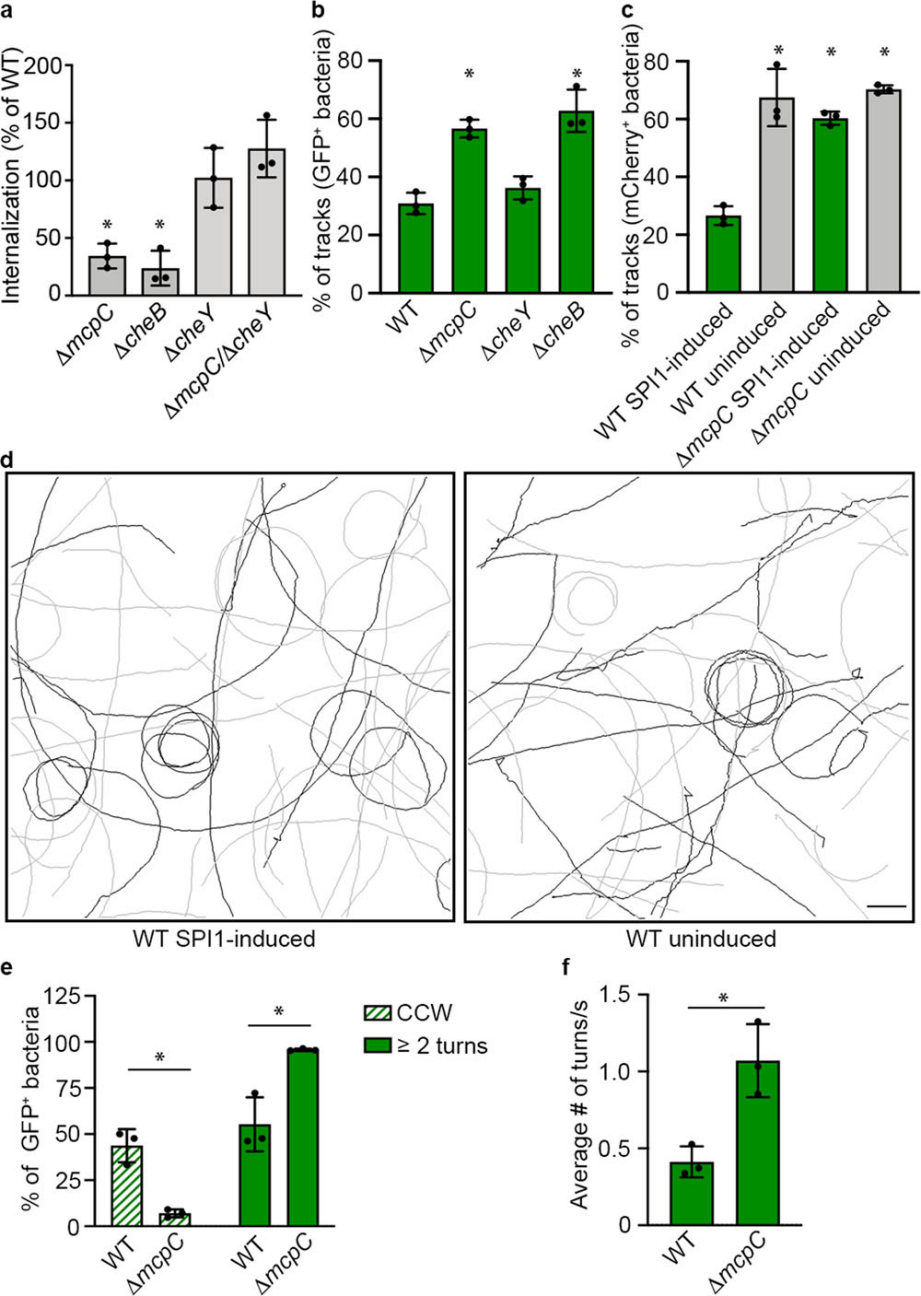
McpC promotes CCW flagellar rotational bias in SPI1-induced bacteria. **a** HeLa cells were infected with the indicated strains for 1.5 h pi. Shown is the mean ± SD inoculum normalized to WT of *n* = 3 independent experiments. Statistical significance was determined by one-way Anova followed by Tukey’s multiple comparisons (WT vs. Δ*mcpC*: *p* = 0.009, WT vs. Δ*cheB*: *p* = 0.003, WT vs. Δ*cheY*: *p* = 0.9, WT vs. Δ*mcpC/*Δ*cheY*: *p* = 0.4). **b**, **c** Swimming behavior of the indicated strains harboring either (**b**) P*prgH*-*gfp*[LVA] or (**c**) dual P*prgH*-*gfp*[LVA]. **b** Shown is the mean ± SD percentage of GFP+ (SPI1-induced) bacteria with tracks containing >2 angle changes for *n* = 3 individual experiments in which 2872 WT bacteria, 2038 Δ*mcpC* bacteria, 947 Δ*cheB* bacteria, and 2654 Δ*cheY* bacteria were examined. Statistical significance was determined by one-way Anova followed by Tukey’s multiple comparisons (WT vs. Δ*mcpC*: *p* = 0.0008, WT vs. Δ*cheY*: *p* = 0.6, WT vs. Δ*cheB*: *p* = 0.0002). **c** Shown is the mean ± SD percentage of tracks containing >2 angle changes for *n* = 3 individual experiments in which 1625 WT SPI1-induced bacteria, 789 WT uninduced bacteria, 907 SPI1-induced Δ*mcpC* bacteria, and 470 Δ*mcpC* uninduced bacteria were examined. Statistical significance was determined by one-way Anova followed by Tukey’s multiple comparisons (WT SPI1-induced vs. WT uninduced: *p* = 0.0001, WT SPI1-induced vs. Δ*mcpC* SPI1-induced: *p* = 0.0003, WT SPI1-induced vs. Δ*mcpC* uninduced: *p* = 0.0001). **d** Representative tracks from SPI1-induced mCherry^+^GFP^+^ WT bacteria (left) and tracks from uninduced mCherry^+^GFP^-^ WT bacteria (right). Gray tracks contain ≤ two angle changes, black tracks contain >2 angle changes. Scale bar, 20 µm. **e**, **f** Flagella tethering assay. SPI1-induced bacteria harboring P*prgH*-*gfp*[LVA] were tethered to glass and imaged. Shown are the mean ± SD % of GFP-positive bacteria that had exclusively CCW rotation (hatched bars) or GFP-positive bacteria that reversed two or more times during the 15 s observation period (solid bars). Data are from *n* = 3 independent experiments in which 65 WT bacteria and 71 Δ*mcpC* bacteria were examined. Statistical significance was determined using two-way Anova followed by Sidak’s multiple comparisons (WT vs Δ*mcpC* exclusively CCW: *p* = 0.002, WT vs Δ*mcpC* with 2+ turns: *p* = 0.009). (**f**) Shown is the mean ± SD number of turns/s for the dataset in **e**. Statistical significance was determined by a two-tailed paired Student’s *t*-test (WT vs. Δ*mcpC*: *p* = 0.02). Green indicates that GFP-positive bacteria were analyzed in the assay. ns = not significant, *P < 0.05. Source data are provided as a Source Data file.

If chemotactic signaling by McpC optimizes invasion, then disruption of all chemotaxis signaling should recapitulate a Δ*mcpC* invasion phenotype. To test this, we constructed a *cheY* mutant, which is unable to promote flagellar motor reversals (locked smooth), and a *cheB* mutant, which overproduces phosphorylated CheY (locked tumbly). Δ*cheY* had no invasion defect in HeLa cells, indicating chemotactic signaling is not required for invasion (Fig. 6a). Conversely, overproduction of phosphorylated CheY in Δ*cheB* resulted in a large invasion defect. Intriguingly, Δ*mcpC*/Δ*cheY* had no defect, indicating that forced smooth swimming can rescue Δ*mcpC* invasion. These results suggest that McpC prevents motor reversals and promotes smooth swimming.

Since Δ*mcpC* has only a slight motility defect on agar swim plates^41^, possibly because of McpC dependence on SPI1 induction (Fig. 2), we used light microscopy to assess flagella rotational bias in SPI1-induced free-swimming bacteria. We used P*prgH-gfp*[LVA] to selectively track and analyze swimming patterns of SPI1-induced GFP^+^ bacteria^42^. Approx. 31% of WT tracks contained more than two angle changes (>30°), while ~57% of Δ*mcpC* tracks did (Fig. 6b). Furthermore, swimming behavior of GFP^+^ WT bacteria was indistinguishable from the Δ*cheY* mutant (smooth), while Δ*mcpC* bacteria were identical to a *cheB* mutant (tumbly) (Fig. 6b). Thus SPI1-induced WT STm have a “locked smooth” swimming phenotype that is dependent on expression of *mcpC*. If this is true, then non SPI1-induced bacteria should not be “locked smooth”. In order to simultaneously track free-swimming GFP^−^ and GFP^+^ bacteria, we added constitutive *mCherry* expression to construct dual P*prgH*-*gfp*[LVA]. Comparison of mCherry^+^/GFP^+^ bacteria (SPI1-induced) vs. mCherry^+^/GFP^−^ bacteria (non-SPI1-induced) revealed that non-SPI1-induced WT bacteria (Fig. 6c, d) behave similarly to the *mcpC* and *cheB* mutants (Fig. 6b). In contrast, SPI1-induced and non-SPI1-induced Δ*mcpC* behaviors were indistinguishable, further confirming the requirement for McpC in this phenotype (Fig. 6c). Surprisingly, these swimming phenotypes were observed in the absence of added ligands, and a high-throughput screen failed to identify any ligands (Supplementary Data 2). Smooth swimming occurs when all motors spin CCW resulting in bundled flagella. Cells tumble when one or more motors switch to CW, resulting in dispersal of the attached filament from the bundle. *E. coli* cells can tumble when one motor switches to CW; however, *Salmonella* has more flagella than *E. coli*^43,44^ and it is unclear whether a single motor reversal in *Salmonella* will cause a tumble or less obvious disruptions to smooth swimming (Fig. 6d). To analyze single motors, we performed flagellar tether assays^45^ using P*prgH-gfp*[LVA] to identify SPI1-induced bacteria. More GFP^+^ WT bacteria had motors displaying exclusively CCW rotation compared to GFP^+^ Δ*mcpC*, which reversed more often (Fig. 6e). Additionally, the frequency of turns in reversing motors was higher in GFP^+^ Δ*mcpC* than GFP^+^ WT (Fig. 6f). Thus, the SPI1 transcriptional program includes a HilD-dependent mechanism to promote unstimulated extended smooth swimming via McpC.

## Discussion

Here we identified a chemoreceptor, McpC, as a critical component in early colonization of the mammalian gut. By promoting smooth swimming in SPI1-induced “invasive” bacteria, McpC maximizes interaction with, and invasion of, the intestinal epithelium in the uninflamed gut. Although flagellar motility exists in both T3SS1-expressing and non-expressing cells, we found that swimming behavior is different for SPI1-induced bacteria (those that go on to colonize host epithelium) vs. non-SPI1-induced bacteria (presumably those that replicate and thrive within the gut lumen). Under SPI1-inducing conditions, HilD derepresses *mcpC* by displacement of H-NS from its promoter region. Production of McpC results in CCW bias and a smooth swimming phenotype that facilitates invasion. In the GI tract, SPI1-induced STm are predominantly located close to the mucosal surface^11,46^. We propose that McpC maximizes the chance of these invasive bacteria interacting with their target cells via a process that involves near surface swimming^47,48^. On the other hand, non-SPI1-induced STm, which do not express McpC, retain the normal chemotactic functions that are required to thrive in the lumen of the inflamed gut^1,2,49–51^.

Smooth swimming is induced when chemoreceptors bind an attractant, and is typically followed by rapid adaptation, mediated by the methyl-transferase/-esterase pair CheBR, ensuring that the bacteria are rapidly reset^52^. However, we were unable to identify a ligand for McpC and indeed, in our experimental assays for swimming behavior and flagellar rotation, no exogenous ligands were required. This suggests that a potential McpC ligand could be an intrinsic molecule or a form of energy sensing. Alternatively, the intrinsic conformational structure of McpC may mimic a ligand bound state. To our knowledge, such a bacterial chemoreceptor has not been identified in nature, however replacement of single residues at key locations in the model chemotaxis receptor Tsr mimic ligand binding, likely through changes in the protein structure^53^. If McpC has acquired such a mutation, repression by H-NS would effectively limit any detrimental effects on chemotaxis in non-SPI1-induced bacteria. In addition, the long-lived signature of McpC-induced smooth swimming indicates a lack of, or poor, adaptation. Indeed, McpC does not contain the conserved NWE^T^/_S_F pentapeptide motif that recruits CheBR, and thus may require “adaptation assistance” by other chemoreceptors^54,55^. Thus, we propose McpC induces smooth swimming via a non-canonical mechanism that is ligand-independent and resistant to adaptation.

It was hypothesized nearly 30 years ago that chemotaxis and invasion would share common regulatory elements in *Salmonella*^3^. Smooth swimming mutants have increased invasion in cultured epithelial cells whereas tumbly mutants, which reverse direction frequently, have reduced invasion^3–5^. Our work has identified McpC as the link between invasiveness and smooth swimming in STm, the expression of which is coordinated with the T3SS1 and flagella by the master regulator HilD. Interestingly, naturally occurring mutations in chemotaxis genes, which potentially further increase smooth swimming, have been identified in the human restricted *Salmonella* Paratyphi A and Typhi as well as in the highly invasive zoonotic serovar Cholerasuis^56,57^. Additionally, smooth swimming mutants of some *Vibrio* species also display hypervirulence^58,59^ and it has been hypothesized that repression of chemotaxis gene expression, leading to smooth swimming, may be responsible for the hypervirulence of *Vibrio cholerae* shed in rice water stools^60,61^. The fact that genetically unrelated enteric pathogens coordinate smooth swimming with invasiveness indicates this may be a more widespread virulence strategy. While the regulatory and functional mechanisms need further study, this is a potential target for the development of novel antimicrobial therapies.

## Methods

### Bacterial cultures and growth conditions

STm SL1344 was used in all experiments (Supplementary Table 1). Strain stocks were frozen in 15% glycerol and stored at −80 °C. Strains were streaked onto LB agar supplemented with appropriate antibiotics and stored at 4 °C for up to 1 week.

#### SPI1-induction

As previously described^12^, SPI1 induction was as follows: Overnight (16–18 h) cultures were prepared by inoculating one colony into 2 mL LB-Miller broth (US Biological, Animal Free) (10 g/L salt) with selective antibiotics, in a loose-capped 14 mL polypropylene round-bottom tube (Becton Dickinson) and incubated at 37 °C with shaking (225 rpm). To induce SPI1, 0.3 mL of the overnight culture was inoculated into 10 mL LB-Miller broth (no antibiotics), in a 125 mL Erlenmeyer flask for 3.5 h at 37 °C with shaking (225 rpm).

#### Animal inoculations

For mouse experiments, bacteria were grown in a 125 mL Erlenmeyer flask in 10 mL LB-Miller broth containing the appropriate antibiotic for 18 h at 37 °C with shaking (225 rpm) and diluted in sterile pharmaceutical grade saline (SPGS). For calf experiments, loose-capped 14 mL polypropylene round-bottom tubes (Becton Dickinson) were used for both overnight cultures and subcultures. One colony was inoculated into 5 mL LB-Miller broth with selective antibiotics and incubated for 14 h at 37 °C with shaking (225 rpm), then diluted 1:100 in 5 mL LB-Miller without antibiotics and sub-cultured for 4 h at 37 °C with shaking (225 rpm). Cultures were diluted in LB-Miller for inoculation.

### Growth curves and transcriptional reporter assays

Overnight cultures of STm were diluted 1:25 and 200 µL was aliquoted in triplicate into 96-well plates and grown in an Infinite 200 Pro plate-reader (Tecan). Plates were shaken at 37 °C. OD600 and fluorescence (Ex 478, Em 515) were read every 15 min. OD600 and fluorescence values from blank media were subtracted from the average of triplicate wells for each sample.

### Mammalian cells

HeLa (human cervical adenocarcinoma, ATCC) cells were grown at 37 °C in 5% CO_2_ in complete growth medium: Eagle’s minimal essential medium (Mediatech) supplemented with 10% (v/v) heat-inactivated fetal bovine serum (FBS) (Thermo Fisher), 2 mM l-glutamine and 1 mM sodium pyruvate. Caco2 subclone C2BBe1 (human colorectal adenocarcinoma, ATCC CRL-2012) were grown in Dulbecco’s minimal essential medium (DMEM) supplemented with 10% (v/v) heat-inactivated FBS, 4 mM l-glutamine and 10 μg/mL human transferrin. Cells were passaged as recommended by ATCC and used for experiments within 15 passages of receipt. Human peripheral blood monocytes, enriched by apheresis, were obtained from peripheral blood provided by the Department for Transfusion Medicine and the National Institutes of Health Clinical Center at the National Institutes of Health (Bethesda, MD). Monocytes were further enriched by centrifugation with Ficoll-Paque (GE Healthcare) and then resuspended in a freezing medium of 10% dimethyl sulfoxide (DMSO)–90% FBS at 10^8^ cells/mL and stored in liquid N_2_. Monocytes were purified with the Dynabeads Untouched human monocytes kit (Thermo Fisher); the purity of recovered cells was routinely >90% CD14+ by flow cytometry. These cells were plated in complete medium containing RPMI 1640 medium (Gibco), 1 mM sodium pyruvate, 1× MEM nonessential amino acids, 10 mM HEPES buffer, 2 mM glutamate, 5% (vol/vol) heat-inactivated human male AB serum (HuS) (Sigma-Aldrich), and 100 ng/mL human recombinant macrophage colony-stimulating factor (M-CSF) (PeproTech). Cells were grown in 5% CO_2_ at 37 °C and used for assays on day 7. On days 3 and 5, 50% of the volume of the cultures was replaced with fresh complete RPMI, 5% serum, and 200 ng/mL M-CSF. All data are from three independent experiments using cells prepared on different days from different donors.

### Construction of chromosomal deletion mutants

The bacteriophage λ Red recombinase system was used for construction of gene disruption mutants in STm^62^. Mutants were verified by PCR analysis, and the mutation transduced by P22 back to WT SL1344. If necessary, antibiotic cassettes were removed using pCP20 before combining mutations using P22 transduction.

### Plasmid construction

All plasmid ligation reactions were carried out using T4 DNA ligase (Promega). Restriction enzymes were obtained from New England Biolabs (NEB). The high-fidelity polymerase Phusion was used for all PCR reactions (NEB). PCR primers were sourced from Integrated DNA technologies. All plasmid constructs were verified by sequencing. Plasmids are listed in Supplementary Table 1. Oligonucleotide sequences are listed in Supplementary Table 2.

### Plasmid backbones

A synthetic transcriptional terminator (Part Bba_B0015 of the Registry of Standard Biological Parts (parts.igem.org)) was cloned into the pWSK29ΔP*lac Kpn*I site using primers B0015 *Kpn*I F and B0015 *Kpn*I R. Likewise, the terminator was added to pMPMA3ΔP*lac* using the *Not*I and *Sac*II sites with the primers B0015 *Not*I F and B0015 *Sac*II R.

### Transcriptional reporters

Promoter regions (positions with respect to start ATG), *mcpC* (−387 to +93) and (−79 to +93) were PCR amplified with P*mcpC* −387 *Xba*I F or P*mcpC* −79 *Xba*I F and *mcpC* stop *Kpn*I R (which introduces a stop codon) and ligated to a *Xba*I/*Kpn*I digest of pGFP[LVA] (Clontech). This produces an operon-like fusion, in which the transcribed mRNA codes for a short *mcpC* peptide prior to a stop codon, followed by a strong ribosome-binding site (RBS) and *gfp*[LVA]. The fusion was then amplified without the LVA tag using primers P*mcpC* −387 *Xba*I F or P*mcpC* −79 *Xba*I F and *gfp* no LVA *Hind*III R and cloned into a *Xba*I/*Hind*III digest of pMPMA3ΔP*lac* TT. Reporter fusions were moved into a plasmid with a compatible origin (*ori* pMB1) to that of the *hns* expressing plasmids (pMPMT6 *ori* p15A) to enable maintenance of both plasmids in STm. To do this, the *mcpC* −387 to +93 *gfp* fusion was amplified with P*mcpC* −387 *EcoR*V F and B0015 *Sac*I R and ligated into an *EcoR*V/*Sac*I digest of pBAD18-Cm, which replaces the P_BAD_ promoter and most of the *araC* gene, resulting in pMB1 P*mcpC* −387 *gfp*. In order to change the sigma factor recognition site, a gene fragment containing the change (Supplementary Table 2) was synthesized (Integrated DNA Technologies) to replace the fragment between *EcoR*V and *Pml*I sites of pMB1 P*mcpC* −387 *gfp*. To construct dual P*prgH*-*gfp*[LVA], the ProC-*mCherry*-TT expression cassette was PCR amplified from pCON1-ProC*.mCherry* and cloned into the *Not*I and *Sac*I sites of pMPMA3ΔP*lac* P*prgH-gfp*[LVA].

### Expression constructs

*mcpC* gene was amplified using P*mcpC* −387 *Xba*I F and *mcpC* ORF *Not*I R and cloned into *Xba*I/*Not*I digested WSK29ΔP*lac* TT, resulting in p*mcpC*. The *inv* locus was excised from pRI203^63^ using *BamH*I and cloned into pWSK29 in the same direction as the P*lac* promoter, resulting in pWSK29-*inv*. The *hilD* gene including its 3′-UTR was PCR amplified from SL1344 genomic DNA using primers *hilD Nhe*I RBS F and *hilD* 3′-UTR *Sph*I R and cloned into the *Nhe*I/*Sph*I sites of a low copy arabinose-inducible expression plasmid (pMPMA3ΔP*lac* p_BAD_) made by subcloning the *araC* gene and promoter from pBAD18-Cm into pMPMA3ΔP*lac* using *Cla*I and *Hind*III.

### Gentamicin protection assay

These assays were done similar to previous studies^64,65^. Briefly, human monocytes were seeded in 96-well plates at 4 × 10^4^ cells per well, differentiated as described above, and used for infection on day 7. Immortalized cell lines were seeded 20–24 h prior to infection in 24-well plates at 4.5 × 10^4^ cells (HeLa) or 5.5 × 10^4^ per well (C2Bbe1). C2Bbe1 were seeded onto collagen-coated plates. Late-log phase cultures (SPI1-induced) STm were collected by centrifugation at 8000×*g* for 2 min, washed and resuspended in Hank’s buffered saline solution (HBSS) and used immediately to infect cells for 10 min at an MOI of ~50 for epithelial cells and ~10 for human macrophages. T3SS-1 mutants were used at an MOI of ~30 in human macrophages. Centrifugation at 500×*g* for 5 min was not used except in Fig. 4. Extracellular bacteria were removed by washing with HBSS and cells were incubated in antibiotic-free complete growth media until 30 min pi. Cells were then incubated for either 15 min (macrophages) or 1 h (epithelial cells) in complete growth media supplemented with l-histidine (500 μg/mL) and gentamicin (50 μg/mL). The media was then replaced by complete growth media supplemented with l-histidine (500 μg/mL) and gentamicin (10 μg/mL) for the remainder of the infection. At indicated time-points, monolayers were lysed in 0.2% (w/v) sodium deoxycholate in PBS and viable intracellular bacteria were enumerated by plating on LB agar. Internalization was calculated as the % of the inoculum remaining after the gentamicin protection assay and normalized to WT.

### Protein purification

MBP-HilD and MBP-H-NS were expressed in *E. coli* BL21/DE3 containing pMAL-*hilD* or pMAL-*hns* and purified by using an amylose column per manufacturer’s instructions (NEB). After purification, the buffer was exchanged to 20 mM Tris–HCl pH 7.5, 1 mM EDTA, 40 mM KCl using a PD-10 desalting column (Amersham). Protein concentration was determined using a BCA assay (Bio-Rad) and proteins were stored in aliquots in this buffer with 50% glycerol and 1 mM DTT at −20 °C. The MBP was cleaved off of MBP–H-NS using Factor Xa protease per manufacturer’s instructions (NEB). Recombinant ligand-binding domain of McpC (amino acid residues 35–189) or Tar (amino acid residues 34–190) including N-terminal 6x His tags on each were commercially purified (GenScript).

### Electrophoretic mobility shift assays (EMSAs)

DNA fragments were PCR amplified from SL1344 genomic DNA using the following primer pairs: P*mcpC* −387 - +93:P*mcpC* −387 XbaI F and P*mcpC* stop *Kpn*I R; P*mcpC* −79 - +93: P*mcpC* −79 *Xba*I F and P*mcpC* stop *Kpn*I R; P*mcpB* −333 - +45:P*mcpB Xba*I F and P*mcpB* stop *Kpn*I R. PCR products were purified using the QIAquick PCR purification kit (Qiagen). Reactions contained 100 ng of PCR product with increasing concentrations of purified MBP-HilD or H-NS in binding buffer containing 10 mM Tris–HCl (pH 8), 50 mM KCl, 1 mM dithiothreitol (DTT), 0.5 mM EDTA, 5% glycerol, and 10 μg/mL bovine serum albumin (BSA), in a total volume of 20 μL. Reactions were incubated for 20 min at RT and then separated by electrophoresis in 6% nondenaturing polyacrylamide gels in 0.5× Tris–borate–EDTA (TBE) buffer. In order to differentiate the size of MBP–HilD–DNA complexes from H-NS–DNA complexes in competitive EMSA gels, MBP was first cleaved from MBP–H-NS using Factor Xa protease (NEB). For competitive EMSAs, the DNA fragment was first incubated with 0.4 μM H-NS for 15 min and then incubated with increasing concentrations of MBP-HilD for an additional 20 min. Gels were stained with SYBR safe (Thermo Fisher) and visualized with an alpha-imager UV transilluminator (Bio-Rad).

### Ligand screening

The dimerization status of purified ligand-binding domain of McpC was assessed commercially using sedimentation velocity analytical ultracentrifugation (SV-AUC) (KBI BioPharma) and revealed that dimers were present at ≥4 mg/mL. Thermal shift assays were done as previously described^66^ using a BioRad CFX Real-Time PCR instrument in a 384-well format. Ligands were prepared by dissolving Biolog PM compounds in 50 μL water to obtain a final concentration of 10–20 mM, except for PM4A which was dissolved in 20 μL. A 10 mM solution of l-cystine was also prepared in 50 mM HCl. Each 10 μL reaction contained 4 mg/mL protein (~212 μM), 10× SYPRO Orange dye (Thermo Fisher) in a 50 mM HEPES buffer pH 8. 2 μL of ligand was added to each well. As a positive control, Tar LBD was used at 10 μM protein with 1 mM l-aspartate. Samples were heated by 1 degree per min from 25–75 °C. Protein unfolding curves were monitored by fluorescence of SYPRO Orange. Melting temperatures were calculated using non-linear fitting to the Boltzmann equation (GraphPad).

### RNA sequencing

Total RNA was quantified using Quant-it RiboGreen assay (Thermo Fisher) and 500 ng total RNA was depleted of ribosomal RNAs using RiboZero bacterial rRNA depletion kit, following the manufacturer’s recommended procedure (Epicentre). Ribosomal-depleted RNAs were purified using RNAClean XP beads (Beckman Coulter Life Sciences) and eluted in Fragment, Prime, and Finish buffer found in Illumina TruSeq Stranded mRNA Library Preparation Kit (Illumina Inc). This kit was then used to generate sequencing libraries beginning at the fragmentation step with no other modifications. Each sample was given a unique molecular barcode and fragment-sized using a BioAnalyzer High Sensitivity chip (Agilent). The samples were quantitated using Kapa Library Quant kit (Illumina) Universal qPCR mix (Kapa Biosystems) and diluted to a 2 nanomolar working concentration. Equal volumes were combined into a single pool and clustered across a RAPID 2-lane flowcell. The flowcell was then sequenced on an Illumina HiSeq 2500 instrument for 60 cycles in one direction with an additional seven cycles to sequence the molecular barcodes, generating an average of 13.5 million reads per sample.

Raw fastq reads were trimmed of Illumina adapter sequencing using a proprietary script and then trimmed and filtered for quality using the FASTX-Toolkit (Hannon Lab, CSHL). Remaining reads were mapped to the *Salmonella enterica* subsp. *enterica* serovar Typhimurium str. SL1344 genome (chromosome and plasmids); NC_016810.1, NC_017718.1, NC_017719.1, and NC_017720.1 using Bowtie2^67^ with parameters --score-min L,0,−0.15. Reads mapping to genes were counted using htseq-count^68^. The Bioconductor package DESeq2^69^ was used for data normalization and differential gene expression analysis. Data were from three independent samples from each strain.

### Immunofluorescence microscopy

#### Cultured cells

HeLa cells were plated on glass coverslips in 24-well plates (5.5 × 10^4^ cells per well). For cell association assays using pWSK29-*inv*, cells were infected as described above except for 6 min instead of 10 min. Infected cells were washed three times with ice-cold HBSS and fixed in 2.5% (w/v) paraformaldehyde (PFA) for 10 min at 37 °C, followed by three washes in PBS. To differentially stain intracellular and extracellular bacteria, monolayers were blocked (but not permeabilized) in 10% normal donkey serum in PBS, then incubated with goat anti-*Salmonella* CSA-1 (KPL) which had been conjugated to Pacific Blue (Thermo Fisher) per manufacturer’s instructions (1:250) to bind extracellular bacteria. Cells were washed three times, then permeabilized with 0.1% (w/v) saponin plus 10% (v/v) normal goat serum in PBS. Cells were then incubated with goat anti-*Salmonella* CSA-1 which had been conjugated to AlexaFluor 488 (Thermo Fisher) per manufacturer’s instructions (1:250) which will now bind both extracellular and intracellular bacteria, along with AlexaFluor 568-conjugated Phalloidin (Thermo-Fisher) (1:40) to stain cellular F-actin. Coverslips were then washed sequentially with PBS and distilled water then mounted on glass slides in a Mowiol 4–88 solution supplemented with 2.5% (w/v) DABCO (Sigma-Aldrich)^65^. The total number of cells with associated bacteria was counted. For evaluation of ruffle size, GFP-expressing bacteria were used to infect cells as described above except with an MOI of ~10 and contact with host cells was synchronized by centrifugation at 500 × *g* for 5 min at 37 °C. Immediately after centrifugation, cells were washed thrice with warm HBSS and fixed in 2.5% PFA as described above. Cells were incubated in 0.1% saponin in PBS followed by staining with AlexaFluor 568-conjugated Phalloidin and mounting as described above. Images were captured with the same gain and exposure for each sample on a DS-Qi2 camera using a ×60 objective on a Nikon Ti2 epifluorescence widefield microscope. Post-acquisition analysis of ruffle size was done using ImageJ software. Ruffle size was determined as follows: Images were converted to 8-bit binary (0, 255) images and a threshold was placed on the phalloidin channel. A circular ROI measuring 150 × 150 pixels was centered on an individual bacterium associated with phalloidin. Ruffles with more than one associated bacterial cell were not measured. Integrated density in the phalloidin channel within the ROI was measured and divided by 255 to calculate the intensity/pixel area.

#### Calf tissue

Flash frozen 6 mm biopsy punches were thawed/fixed in ice-cold Carnoy’s buffer (60% MeOH, 30% acetic acid, 10% acetic acid) for 3 h on ice before washing as follows: 2× MeOH washes, 30 min each, 2× ETOH washes, 20 min each, 2× PBS washes, 20 min each. Tissues were then embedded and frozen in OCT media and 5 μm sections were cut and placed on slides for staining. Tissue sections were blocked with 2% donkey serum, 1% BSA, 0.1% Triton X-100, 0.05% Tween-20 in PBS, following by staining with the following primary antibodies: mouse anti-cytokeratin clone C-51 (Thermo Fisher, 1:100), rabbit anti-mCherry polyclonal (Thermo Fisher, 1:100), and goat anti-*Salmonella* CSA-1 (1:100) conjugated to AlexaFluor 488 (Thermo Fisher) per manufacturer’s instructions. Secondary antibodies (1:500) were AlexaFluor 568-conjugated donkey anti-rabbit (Thermo Fisher) and CF^®^633 conjugated donkey anti-mouse (Biotium). After staining, slides were stained for 10 min with wheat germ agglutinin (WGA) conjugated to CF^®^405 (Biotium, 1:500), followed by fixation with 2.5% PFA for 10 min. Samples were mounted with ProLong Gold with DAPI (Thermo Fisher). Images were captured using a Zeiss LSM 710 confocal laser-scanning microscope with either a Plan APOCHROMAT ×63/1.4 N.A. objective or a ×20/0.8 N.A. objective.

### Tethered bacteria

Flagellar rotation patterns were determined by a cell tethering assay^70^. SPI1-induced bacteria were passed through a 27-gauge needle ~50× to shear flagella, followed by two washes in motility buffer (10 mM potassium phosphate pH 7, 0.1 mM EDTA). Bacteria were then diluted to ~1.5 × 10^8^/mL in tethering buffer (10 mM potassium phosphate pH 7, 0.1 mM EDTA, 10 mM sodium lactate, 75 mM sodium chloride, and 0.1 mM l-methionine). 0.1 mL of bacterial suspension was mixed with 2 µl of anti-H*i* antiserum (SSI Diagnostica) and placed in black glass bottom 24-well plates for 10 min at RT. Unbound bacteria were removed, and fresh tethering buffer was placed in the wells. 15 s movies were captured with either a Nikon Ti or Ti2 epifluorescence widefield microscope using a Plan Apo VC ×60/1.4 N.A. objective and an Orca Flash 4.0 camera (Hamamatsu) at 200 frames per second (fps). Movies were sub-stacked to 100 fps and exported for spot detection and tracking analysis in Imaris 9.5.0 software. *XY* position information for each track was exported and data was processed using in-house python script. Spin direction of tethered bacteria from frame *j* to frame *j* + 1 was determined by calculating the cross-product of the two positional vectors $\vec R$ with the given *x* and *y* coordinates of each vector as in Eq. (1):

1 $$\vec R_j \times \vec R_{j + 1} = \big( {R_j^x \ast R_{j + 1}^y} \big) - \big( {R_j^y \ast R_{j + 1}^x} \big)$$

The sign of the resultant cross product indicates direction the bacteria is spinning. A direction change was scored if the direction of spin changed from one frame to the next. Direction changes per second were calculated as the total number of direction changes divided by the total number of frames in movie for each bacterium, multiplied by frames per second of movie.

### Swimming pattern analysis

SPI1-induced cultures were diluted 1:500 into tethering buffer, mixed gently and 100 μL/well was placed in black 96-well glass bottom plates. 15 s movies were captured with a Nikon Ti2 epifluorescence widefield microscope using a Plan Fluor Ph2 DLL ×40/0.75 N.A. objective and an Orca Flash 4.0 camera (Hamamatsu) at 30 fps. For simultaneous capture of GFP and mCherry, a DC2 beam splitter (Photometrics) was used with two iXon Ultra cameras (Andor). Individual bacteria were tracked using Imaris 9.5.0. *XY* position data was exported and used to calculate the angle of direction change from frame *j* to *j* + 1. The velocity vector $\vec V$ for each frame *j* containing two components is Eq. (2):

2 $$\vec V_j = \left[ {V_j^x,V_j^y} \right]$$

where each instantaneous velocity components for $\vec V_j$ are calculated from the positional vectors $\vec R$ of the neighboring frames as in Eqs. (3) and (4):

3 $$V_j^x = \frac{{{\mathrm{{d}}}R_j^x}}{{{\mathrm{{d}}}t}} = \frac{2}{{3T}}\big( {R_{j + 1}^x - R_{j - 1}^x} \big) - \frac{1}{{12T}}\big( {R_{j + 2}^x - R_{j - 2}^x} \big)$$

4 $$V_j^y = \frac{{{\mathrm{{d}}}R_j^y}}{{{\mathrm{{d}}}t}} = \frac{2}{{3T}}\big( {R_{j + 1}^y - R_{j - 1}^y} \big) - \frac{1}{{12T}}\big( {R_{j + 2}^y - R_{j - 2}^y} \big)$$

The angle in degrees by which the bacterium changes direction between frames *j* and *j* + 1 is then calculated in Eq. (5):

5 $$\theta ^ \circ = {\mathrm{cos}}^{ - 1}\left( {\frac{{\left( {V_j^x {\,}*{\,} V_{j + 1}^x} \right) + \left( {V_j^y {\,}*{\,} V_{j + 1}^y} \right)}}{{\parallel V_j\parallel * \parallel V_{j + 1}\parallel }}} \right) \ast \frac{{180}}{\pi }$$

where $\parallel {\!}V_j{\!}\parallel$ and $\parallel{\!} V_{j + 1}{\!}\parallel$ are the magnitudes of velocity vector $\vec V_j$ and $\vec V_{j + 1}$. Strains were compared by calculating the percentage of tracks with two or more angle changes of at least 30° each. Each experiment compiled results from five movies per strain.

### Flow cytometry

10–20 μL bacteria were fixed in 500 μL 2.5% (w/v) paraformaldehyde at RT for 10 min, centrifuged and finally washed once in PBS. Bacteria were then stained with 10 μM Syto41 (Thermo Fisher) in PBS for 30 min at RT, washed once with PBS by centrifugation, and resuspended in 1 mL PBS for analysis on a BD LSR II flow cytometer (BD Bioscience). Data were analyzed using FlowJo software (Tree Star). Samples were gated on Syto41^+^ events and the % and mean intensity of GFP^+^ events was measured. Gating strategy is shown in Supplementary Information.

### Mouse experiments

The C57BL/6 mice used in this study were either from a colony of mice originally purchased from The Jackson Laboratory (Bar Harbor, ME) and maintained at the Rocky Mountain Laboratories or purchased from The Jackson Laboratory and used shortly after arrival. Animal facilities maintained the following parameters: temperature, 72 ± 3°F; humidity, 50 ± 10%; dark/light cycle, 12:12 h.

For  oral infection, mice were streptomycin treated 24 h before infection, using a blunt end gavage needle with 100 µl SPGS containing 200 mg/mL streptomycin. Mice were fasted for 4 h prior to all gavages. Mice were gavaged or infected intraveneously by retroorbital injection with a volume of 100 µl. Mice were euthanized by isoflurane inhalation followed by exsanguination. Tissues were collected in screwcap tubes containing 500 µl SPGS and 3–4 2.0 mm zirconia beads (BioSpec Products) and homogenized using a Bead Mill 24 (Fisher Scientific, 4.85 m/s for 20 s). Tubes were weighed before and after organ collection. CFUs were estimated by 10 µl spot plating of 10-fold dilutions on LB agar plates containing the appropriate antibiotic.

### Bovine ligated jejunal and ileal loop surgeries

The ligated jejunal and ileal loop proceedures were similar to previous studies with some important changes^11^. Calves 4–6 weeks of age were obtained from the Texas A&M University Veterinary Medical Park and received colostrum prior to isolation. Animals were fed antibiotic-free milk replacer twice daily and water ad libitum. Prior to surgery, calves were tested for *Salmonella* spp. in fecal excretions. Calves were fasted 12 h before surgery. After laparotomy, the distal jejunum and ileum were externalized and loops, ~6 cm in length, were formed with 1-cm spacer loops in-between. Bacterial cultures of 1 mL LB-Miller containing 10^7^ total CFU were prepared as described above and loaded into a 3 mL syringe with a 26-gauge needle and kept on ice until inoculation into the loop via intraluminal injection. Following inoculation, the loops were returned to the body cavity and maintained at ~37 °C. At 2 h pi, loops were excised and processed. Briefly, loops were weighed and opened, and luminal fluid collected. Mucus was collected by gently stamping a 10 mm circular filter paper onto the tissue surface before immersion of the filter into SPGS. Six mm tissue biopsy punches were either flash frozen or placed into SPGS containing 100 μg/mL gentamicin for 1 h before washing twice and placing into fresh SPGS. All samples were collected or ultimately placed into screwcap tubes containing SPGS and 3–4 2.0 mm zirconia beads (BioSpec Products) and homogenized using a Bead Mill 24 (Fisher Scientific, 4.85 m/s for 20 s). Tubes were weighed before and after sample collection and CFUs were estimated by plating on LB agar plates containing the appropriate antibiotic. Loops from three independent calves were utilizedy.

### Reporting summary

Further information on research design is available in the Nature Research Reporting Summary linked to this article.

## Supplementary information

Supplementary Information

Peer Review File

Description of Additional Supplementary Files

Supplementary Data 1

Supplementary Data 2

Reporting Summary

## Data Availability

The RNA sequencing data has been deposited in NCBI’s Gene Expression Omnibus and are accessible at https://www.ncbi.nlm.nih.gov/geo/query/acc.cgi?acc=GSE156765. All other data supporting the findings of this study are available within the paper and the Supplementary Information. Source data are provided with this paper.

## Source data

Source Data
