# Supplementary material for: Regulatory protein HilD stimulates Salmonella Typhimurium invasiveness by promoting smooth swimming via the methyl-accepting chemotaxis protein McpC
Source: Nat Commun. 2021 Jan 13;12:348. doi: 10.1038/s41467-020-20558-6 (PMC7806825; doi:10.1038/s41467-020-20558-6)
Supplement: Supplementary file 1 — Supplementary Information [file 41467_2020_20558_MOESM1_ESM.pdf]

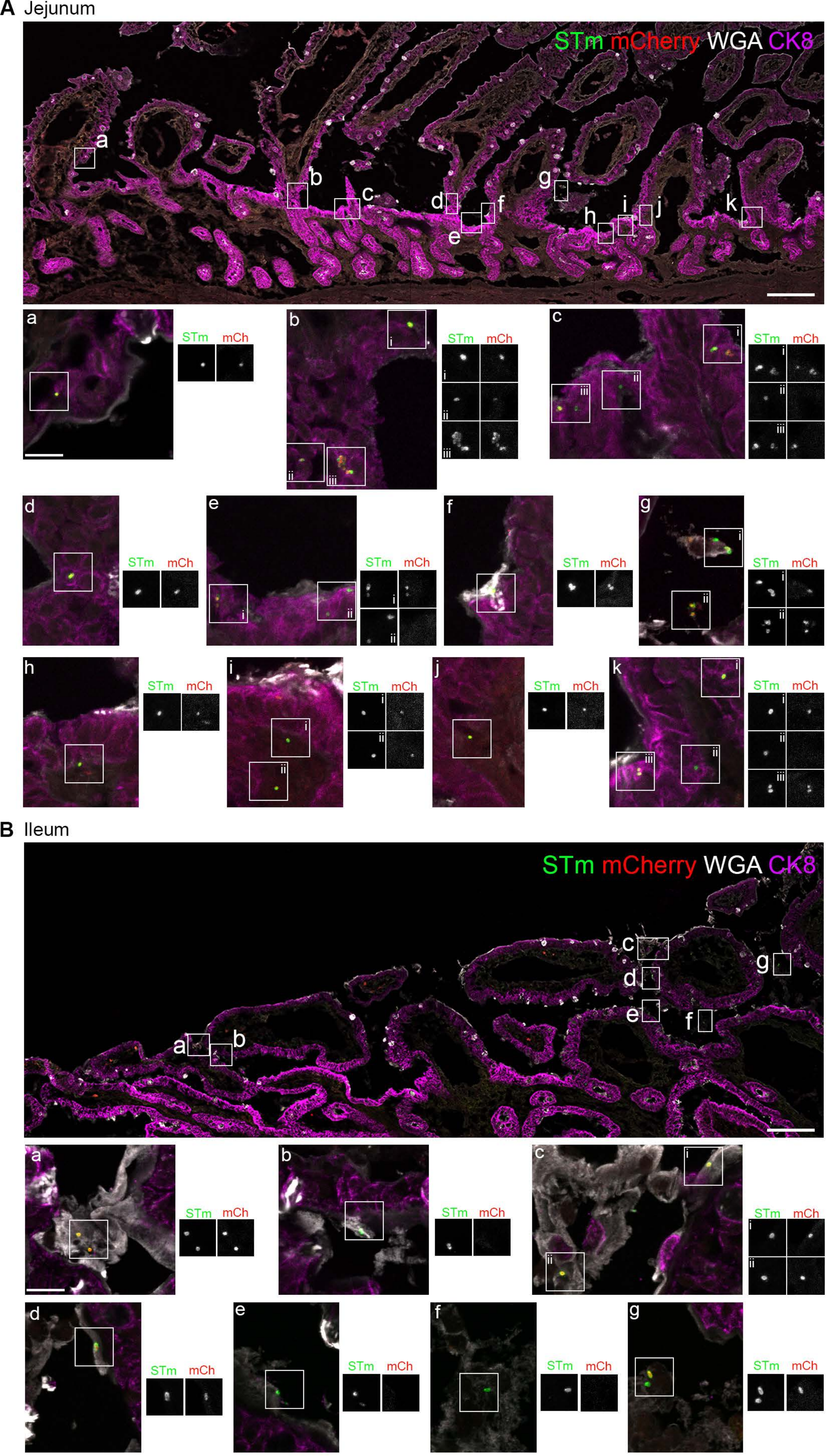

Supplementary Figure 1. Microscopy Analysis of Calf Tissue – WT (mCherry^{+}) and Δ*mcpC* (mCherry^{-}). Representative infected calf jejunal (a) or ileal (b) loop biopsy sections (5 μM) from the same loops as Figure 5b. Loops were infected 1:1 with WT (mCherry^{+}) and Δ*mcpC* (mCherry^{-}). Shown are maximum intensity projections of tissue stained with anti-CSA (green), anti-mCherry (red), wheat germ agglutinin (WGA, white), and anti-cytokeratin-8 (magenta). The images were taken at 20x magnification with the 100 μM scale bar shown. Magnified 63x insets of boxed regions show single channel images of single bacteria stained with anti-CSA (total) and/or anti-mCherry (WT bacteria) with a 10 μM scale bar shown in each panel a. Results were similar for the other loops.

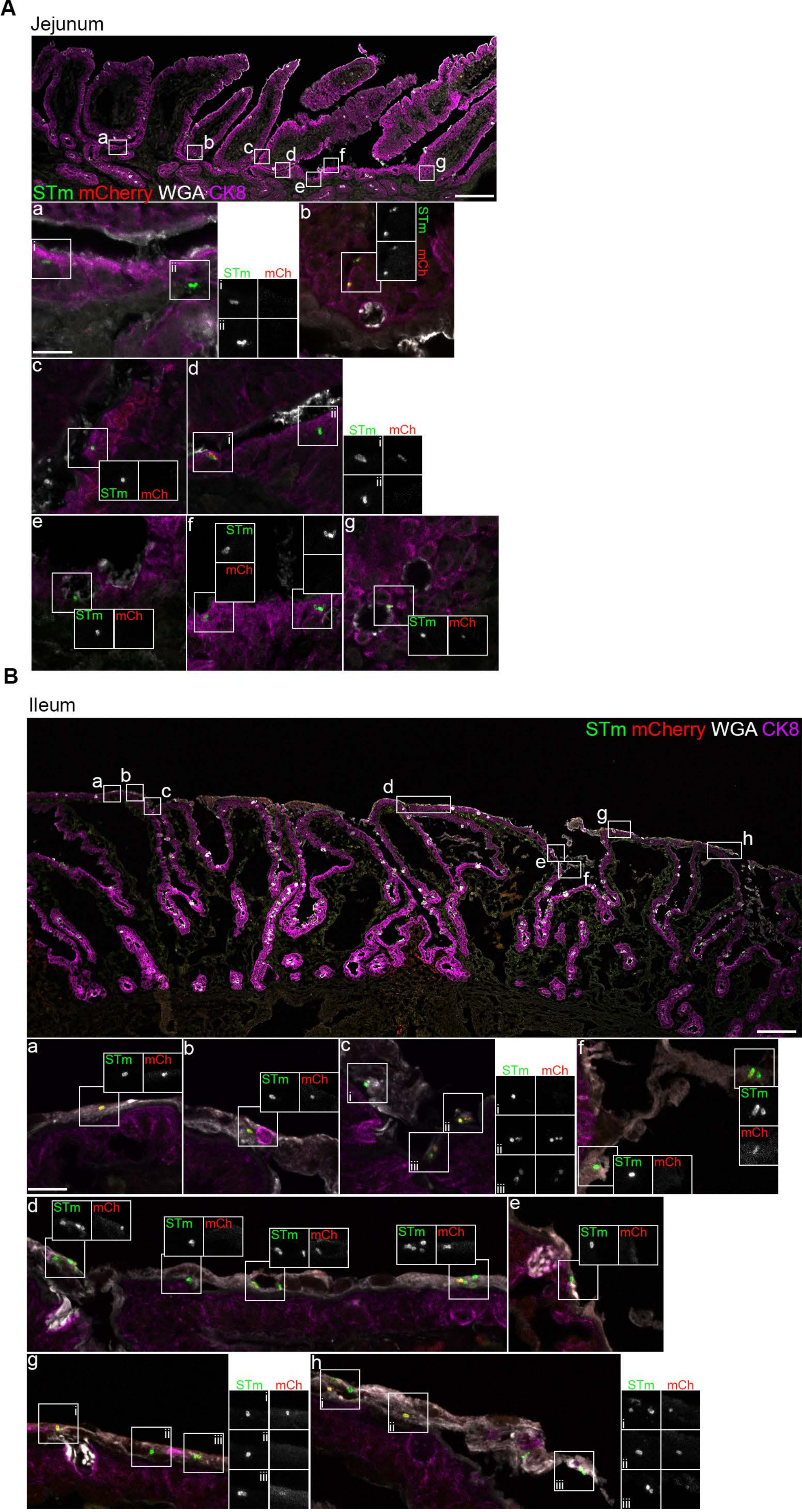

Supplementary Figure 2. Microscopy Analysis of Calf Tissue – WT (mCherry$^-$) and Δ*mcpC* (mCherry$^+$). Representative infected calf jejunal (a) or ileal (b) loop biopsy sections (5 μM) from the same loops as Figure 5b. Loops were infected 1:1 with WT (mCherry$^-$) and Δ*mcpC* (mCherry$^+$). Shown are maximum intensity projections of tissue stained with anti-CSA (green), anti-mCherry (red), wheat germ agglutinin (WGA, white), and anti-cytokeratin-8 (magenta). The images were taken at 20x magnification with the 100 μM scale bar shown. Magnified 63x insets of boxed regions show single channel images of single bacteria stained with anti-CSA (total) and/or anti-mCherry (Δ*mcpC* bacteria) with a 10 μM scale bar shown in each panel a. Results were similar for the other loops.

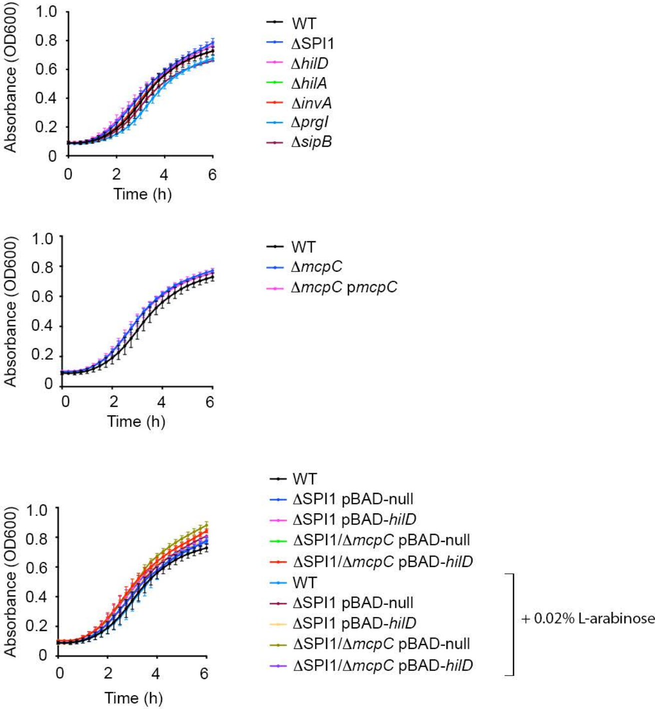

Supplementary Figure 3. Growth curve analysis of indicated strains. Strains were inoculated 1:25 into LB-Miller broth and grown with shaking at 37°C in a platereader. OD600 was taken every 15 min. Wells with media alone were subtracted from average of triplicate wells for each strain. Shown is the mean±SD of *n* = 3 independent experiments.

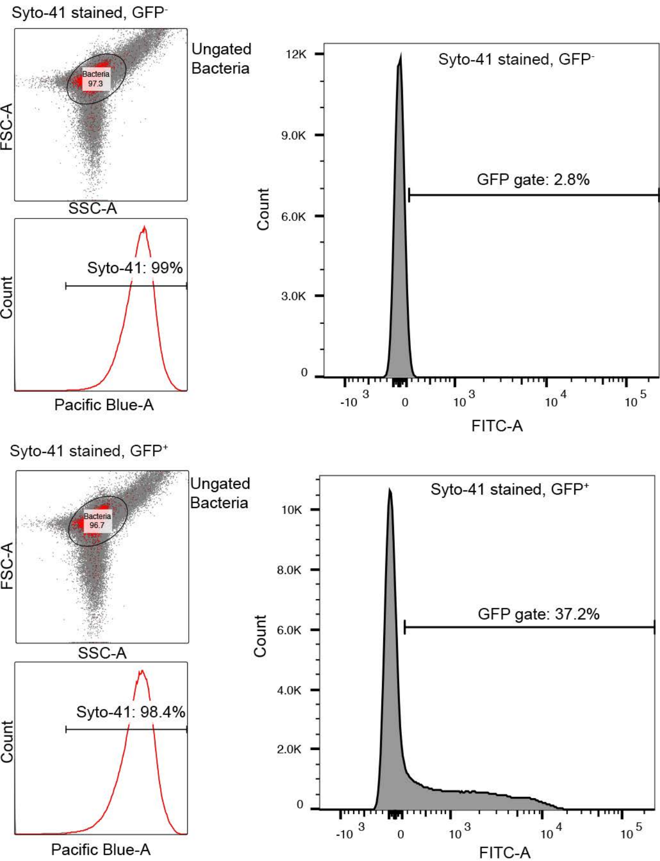

Supplementary Figure 4. Gating strategy for bacterial flow cytometry. Bacteria were fixed and stained with Syto-41. Events were gated on Syto-41 populations and then % of GFP positive events were identified as shown.

Strains and Plasmids	Genotype or Features	Reference
**Strains**		
SL1344	*hisG46, xyl*	[1]
ΔSPI1	SPI1(*sprB* through *invH*)::kan	[2]
Δ*hilD*	*hilD*::kan	[3]
Δ*hilA*	Δ*hilA* (KM011)	[4]
Δ*invA*	*invA*::kan	[5]
Δ*sipB*	*sipB*::kan	[5]
Δ*prgI*	*prgI*::kan	This study
*hilD*Δ3'UTR	*hilD* 3'UTR::FRT	This study
Δ*mcpC*	*mcpC*::cm	This study
Δ*aer*	*aer*::kan	This study
Δ*cheY*	*cheY*::cm	This study
Δ*cheB*	*cheB*::kan	This study
*flhD::*Tn10	Tn10 insertion in *flhD* moved by P22 into SL1344	[6], This study
Δ*fliA*	fliA::kan	This study
**Plasmids**		
pWSK29	pSC101 *ori*, P*lac* promoter	[7]
pWSK29ΔP*lac*	pWSK29 without P*lac* promoter	[8]
pWSK29ΔP*lac* TT (pnull)	pWSK29ΔP*lac* with synthetic transcriptional terminator	This study
pRI203	pBR325 + *Yersinia pseudotuberculosis Inv* locus	[9]
pMPMA3ΔP*lac*	p15A *ori,* pMPMA3 without *lac* promoter	[10]
pMPMA3ΔP*lac* P*prgH-gfp*[LVA]	pMPMA3ΔP*lac* with *prgH-gfp*[LVA] reporter	[10]
pCON1-ProB-*mCherry*	ColE1 *ori*, Constitutive expression of *mCherry*	[8]
pCON1-ProC-*mCherry*	ColE1 *ori*, Constitutive expression of *mCherry*	[8]
dual P*prgH-gfp*[LVA]	Dual *prgH-gfp*[LVA] with ProC-*mCherry*	This study
pMPMA3ΔP*lac* TT	pMPMA3ΔP*lac* with synthetic transcriptional terminator	This study
pWSK29-*inv*	pWSK29 + *Yersinia pseudotuberculosis inv* locus	This study
pBAD18-Cm	pMB1 *ori*, arabinose-inducible expression plasmid	[11]
pMPMA3ΔP*lac* p_{BAD}	P15A *ori*, arabinose-inducible expression plasmid	This study
pMPMA3ΔP*lac* p_{BAD}-*hilD*	pMPMA3ΔP*lac* with arabinose-inducible *hilD* gene including 3'UTR	This study
pWSK-*mcpC* (p*mcpC*)	pWSK29ΔP*lac* TT with P*mcpC-mcpC*	This study
pMAL-*hilD*	pMALc5x + *hilD*	This study
pMAL-*hns*	pMALc5x + *hns*	This study
pMPMA3ΔP*lac* P*mcpC* -387 *gfp*	p15A *ori,* pMPMA3ΔP*lac* TT, transcriptional gfp fusion to P*mcpC* -387 to +93	This study
pMPMA3ΔP*lac* P*mcpC* -79 *gfp*	p15A *ori,* pMPMA3ΔP*lac* TT, transcriptional gfp fusion to P*mcpC* -79 to +93	This study
pMB1 P*mcpC* -387 *gfp*	pMB1 *ori,* transcriptional gfp fusion to P*mcpC* -387 to +93	This study
pMB1 P*mcpC* σ70 *gfp*	pMB1 *ori,* transcriptional gfp fusion to P*mcpC* -387 to +93, sigma 70	This study
pMPMT6 *hns* WT	p15A *ori,* pMPMT6, arabinose inducible *hns*	[3]
pMPMT6 *hns* Q92am	p15A *ori,* pMPMT6, arabinose inducible dominant negative *hns*	[3]

Supplementary Table 1. Strains and Plasmids used in this study.

Supplementary Table 2. Oligonucleotides and DNA sequences used in this study. Lowercase = restriction sites; italics = introduced stop codons; bold = hybridization to lambda red template plasmids PKD3/4; underline = ribosomal binding site; wavy underline = -35 and -10 boxes.

Primer name	Nucleotide sequence (5'→3')	Used for
hilD NheI RBS F	NNNgctagcAGGAGGAATTAACCATGGAAAATGTAACCTTTGTAAGTA	pMPMA3ΔP*lac* P_{BAD}-*hilD*
hilD 3'UTR *Sph*I R	NNNgcatgcAATAAAATGCCGGCCTTAATCC	pMPMA3ΔP*lac* P_{BAD}-*hilD*
B0015 *Kpn*I F	NNNNNNggtaccCCAGGCATCAAATAAAACGAAA	pWSK29ΔP*lac* TT
B0015 *Kpn*I R	NNNNNNggtaccTATAAACGCAGAAAGGCCC	pWSK29ΔP*lac* TT
B0015 *Not*I F	NNNNNNgcggccgcCCAGGCATCAAATAAAACGAAA	pMPMA3ΔP*lac* TT
B0015 *Sac*II R	NNNccgcggTATAAACGCAGAAAGGCCC	pMPMA3ΔP*lac* TT
B0015 *Sac*I R	NNNgagctcTATAAACGCAGAAAGGCCC	pMB1 P*mcpC*-387 *gfp*
P*mcpC* -387 *Xba*I F	NNNtctagaGTTTATTTGTAGGTAGCAATG	pMPMA3ΔP*lac* P*mcpC*-387 *gfp*
P*mcpC* -79 *Xba*I F	NNNtctagaGCGATCAATATCATAAAGTTAGTA	pMPMA3ΔP*lac* P*mcpC*-79 *gfp*
mcpC stop *Kpn*I R	NNNggtacc*TTA*CAAAGACAGAGCGGAACTCAC	P*mcpC* reporters
gfp no LVA *Hind*III R	NNNaagcttTTATTTGTATAGTTCATCCATGCCATGTGTA	pWSK-*mcpC*
mcpC ORF *Not*I R	NNNgcggccgcTTAAGCGGGCTGCGTGTC	pWSK-*mcpC*
P*mcpC* -387 *EcoR*V F	NNNgatatcGTTTATTTGTAGGTAGCAATG	pMB1 P*mcpC*-387 *gfp*
P*mcpB Xba*I F	NNNtctagaGACAGATTGACGCGGCATAA	EMSA frag
P*mcpB* stop *Kpn*I R	NNNggtacc*TTA*CGTCAGCATGACCATACGAATAG	EMSA frag
*hilD Nde*I 1F	NNNcatatgATGGAAAATGTAACCTTTGTAAGTA	pMAL-*hilD*
*hilD Sbf*I 1R	NNNcctgcaggTTAATGGTTCGCCATTTTTATGA	pMAL-*hilD*
*hns Nco*I 1F	CTAccatggGCGAAGCACTTA	pMAL-*hns*
*hns Sbf*I 1R	NNNcctgcaggTTATTGCTTGATCAGGAAAT	pMAL-*hns*
ProSeries *Not*I F	NNNNNNgcggccgcCACAGCTAACACCACGTC	pDUAL-P*prgH*
**Construction of deletion mutants**		
prgI KO F	ACTTTAATTTAACGTAAATAAGGAAGTCATTATGGCAACACC**TGTAGGCTGGAGCTGCTTC**	
prgI KO R	CTGCCCTATAACGGCATTCTCAGGGACAATAGTTGCAATCGA**CATATGAATATCCTCCTTAG**	
hilD 3'UTR KO F	AACTACGCCATCGACATTCATAAAAATGGCGAACCATTAA**TGTAGGCTGGAGCTGCTTC**	
hilD 3'UTR KO R	AATAAAATGCCGGCCTTAATCCACAGGGTTAAAGCCGG**CATATGAATATCCTCCTTAG**	
mcpC KO F	ATGTTTTTGCATAACATTAAAATACGTTCAAAATTATTTATGGCCTTTG**TGTAGGCTGGAGCTGCTTC**	
mcpC KO R	ATTAAGCGGGCTGCGTGTCCTCTTCGCGGACGCGGAACACGTTAAC**CATATGAATATCCTCCTTAG**	
cheY KO 1F	ACAGGCGATACGTATTTGAACCAGGAGTAGTATTTTATGGCGGAT**TGTAGGCTGGAGCTGCTTC**	
cheY KO 1R	GCCTTCATCAGCAGGCTTGATAGATGGTTGCATCATCATCGCATCC**CATATGAATATCCTCCTTAG**	
cheB KO 1F	TGCGCGGACAGACGGTGTATGCGCTAAGTAAGGATAAAGC**TGTAGGCTGGAGCTGCTTC**	
cheB KO 1R	AATTTAAGCTCTTTATCCGCCATAAAATACTACTCCTGGT**CATATGAATATCCTCCTTAG**	
fliA KO 1F	TAATCATGCCGATAACTCATTTAACGCAGGGCTGTTTATC**TGTAGGCTGGAGCTGCTTC**	
fliA KO 1R	ATACGTTGTGCGGCACTTTTCGGGTGCGATCATGCGCGAC**CATATGAATATCCTCCTTAG**	
**Changing P*mcpC* sigma factor**		pMB1 P*mcpC* σ70 *gfp*
P*mcpC* sequence (σ28)	TAAAGTTAGTAACATTATTGCCGATAA	
P*mcpC* sequence (σ70)	TTTACGTAGTAACATTATTGCCGTATGAT	

1. Hoiseth, S. K. & Stocker, B. A. Aromatic-dependent Salmonella typhimurium are non-virulent and effective as live vaccines. *Nature* **291,** 238–239 (1981).
2. Drecktrah, D., Knodler, L. A., Ireland, R. & Steele-Mortimer, O. The mechanism of Salmonella entry determines the vacuolar environment and intracellular gene expression. *Traffic* **7,** 39–51 (2006).
3. Bustamante, V. H. *et al.* HilD-mediated transcriptional cross-talk between SPI-1 and SPI-2. *Proc. Natl. Acad. Sci. U.S.A.* **105,** 14591–14596 (2008).
4. Main-Hester, K. L., Colpitts, K. M., Thomas, G. A., Fang, F. C. & Libby, S. J. Coordinate regulation of Salmonella pathogenicity island 1 (SPI1) and SPI4 in Salmonella enterica serovar Typhimurium. *Infect. Immun.* **76,** 1024–1035 (2008).
5. Finn, C. E., Chong, A., Cooper, K. G., Starr, T. & Steele-Mortimer, O. A second wave of Salmonella T3SS1 activity prolongs the lifespan of infected epithelial cells. *PLoS Pathog.* **13,** e1006354 (2017).
6. Kutsukake, K., Ohya, Y., Yamaguchi, S. & Iino, T. Operon structure of flagellar genes in Salmonella typhimurium. *Mol. Gen. Genet.* **214,** 11–15 (1988).
7. Wang, R. F. & Kushner, S. R. Construction of versatile low-copy-number vectors for cloning, sequencing and gene expression in Escherichia coli. *Gene* **100,** 195–199 (1991).
8. Cooper, K. G., Chong, A., Starr, T., Finn, C. E. & Steele-Mortimer, O. Predictable, Tunable Protein Production in Salmonella for Studying Host-Pathogen Interactions. *Front Cell Infect Microbiol* **7,** 475 (2017).
9. Isberg, R. R., Voorhis, D. L. & Falkow, S. Identification of invasin: a protein that allows enteric bacteria to penetrate cultured mammalian cells. *Cell* **50,** 769–778 (1987).
10. Ibarra, J. A. *et al.* Induction of Salmonella pathogenicity island 1 under different growth conditions can affect Salmonella-host cell interactions in vitro. *Microbiology (Reading, Engl.)* **156,** 1120–1133 (2010).
11. Guzman, L. M., Belin, D., Carson, M. J. & Beckwith, J. Tight regulation, modulation, and high-level expression by vectors containing the arabinose PBAD promoter. *J. Bacteriol.* **177,** 4121–4130 (1995).
