# Supplementary material for: Regulatory protein HilD stimulates Salmonella Typhimurium invasiveness by promoting smooth swimming via the methyl-accepting chemotaxis protein McpC
Source: Nat Commun. 2021 Jan 13;12:348. doi: 10.1038/s41467-020-20558-6 (PMC7806825; doi:10.1038/s41467-020-20558-6)
Supplement: Supplementary file 3 — Description of Additional Supplementary Files [file 41467_2020_20558_MOESM3_ESM.pdf]

Description of Additional Supplementary Files

File Name: Supplementary Data 1
Description: Full RNAseq results comparing WT vs *hilD*D3’UTR. A positive log fold change indicates expression is up in *hilD*D3’UTR compared to WT.

File Name: Supplementary Data 2
Description: Ligand Screening by Thermal Shift Assay. See Methods for details.
