# Supplementary material for: Regulatory protein HilD stimulates Salmonella Typhimurium invasiveness by promoting smooth swimming via the methyl-accepting chemotaxis protein McpC
Source: Nat Commun. 2021 Jan 13;12:348. doi: 10.1038/s41467-020-20558-6 (PMC7806825; doi:10.1038/s41467-020-20558-6)
Supplement: Supplementary file 6 — Reporting Summary [file 41467_2020_20558_MOESM6_ESM.pdf]

nature research

Corresponding author(s): Olivia Steele-Mortimer

Last updated by author(s): Nov 11, 2020

Reporting Summary

Nature Research wishes to improve the reproducibility of the work that we publish. This form provides structure for consistency and transparency in reporting. For further information on Nature Research policies, see Authors & Referees and the Editorial Policy Checklist .

Statistics

For all statistical analyses, confirm that the following items are present in the figure legend, table legend, main text, or Methods section.

n/a	Confirmed	
☐	☒	The exact sample size (*n*) for each experimental group/condition, given as a discrete number and unit of measurement
☐	☒	A statement on whether measurements were taken from distinct samples or whether the same sample was measured repeatedly
☐	☒	The statistical test(s) used AND whether they are one- or two-sided<br>*Only common tests should be described solely by name; describe more complex techniques in the Methods section.*
☐	☒	A description of all covariates tested
☐	☒	A description of any assumptions or corrections, such as tests of normality and adjustment for multiple comparisons
☐	☒	A full description of the statistical parameters including central tendency (e.g. means) or other basic estimates (e.g. regression coefficient) AND variation (e.g. standard deviation) or associated estimates of uncertainty (e.g. confidence intervals)
☐	☒	For null hypothesis testing, the test statistic (e.g. *F*, *t*, *r*) with confidence intervals, effect sizes, degrees of freedom and *P* value noted<br>*Give P values as exact values whenever suitable.*
☒	☐	For Bayesian analysis, information on the choice of priors and Markov chain Monte Carlo settings
☐	☒	For hierarchical and complex designs, identification of the appropriate level for tests and full reporting of outcomes
☒	☐	Estimates of effect sizes (e.g. Cohen's *d*, Pearson's *r*), indicating how they were calculated

Our web collection on statistics for biologists contains articles on many of the points above.

Software and code

Policy information about availability of computer code

Data collection	Nikon Elements AR v5.2, Zeiss ZEN v.2.3 SP1, Bowtie2 v2.2.4, DESeq2 v1.26.0
Data analysis	Imaris v9.5.1, Fiji v2.0.0, FlowJo v10, Prism v8.4.3, Microsoft Excel v16.4.2, Anaconda Python 3.7

For manuscripts utilizing custom algorithms or software that are central to the research but not yet described in published literature, software must be made available to editors/reviewers. We strongly encourage code deposition in a community repository (e.g. GitHub). See the Nature Research guidelines for submitting code & software for further information.

Data

Policy information about availability of data

All manuscripts must include a data availability statement. This statement should provide the following information, where applicable:

- Accession codes, unique identifiers, or web links for publicly available datasets
- A list of figures that have associated raw data
- A description of any restrictions on data availability

The RNA sequencing data has been deposited in NCBI's Gene Expression Omnibus and are accessible at https://www.ncbi.nlm.nih.gov/geo/query/acc.cgi?acc=GSE156765. All other data supporting the findings of this study are available within the paper, the Data Source file and the Supplementary Information.

Field-specific reporting

Please select the one below that is the best fit for your research. If you are not sure, read the appropriate sections before making your selection.

Life sciences study design

All studies must disclose on these points even when the disclosure is negative.

Sample size	Sample size calculations were not performed. Calf ileal loop study used n=4 calves based on published studies, including Raffatellu et al., (DOI: 10.1128/IAI.73.1.146-154.2005), Winter et al., (DOI: 10.1038/nature09415), and Laughlin et al., (DOI: 10.1128/mBio.00946-13). Mouse CI experiments used groups of n=10. We chose n=10 based on the level of variability we see in colonization of mice using oral gavage at early times post infection and have published in Nilsson et al., (DOI: 10.1371/journal.pone.0215190). All experiments used both males and females. CI approach was used to reduce animal group sizes compared to doing single infections.
Data exclusions	No data was excluded.
Replication	All in vitro experiments were repeated at least three times with the n number shown, and all attempts at replication were sucessful. For in vivo experiments, three independent calves were used and each calf had two loops for each CI. Mouse experiments were done once with 10 animals per group, except for Figure 5a, in which the WT:delta mcpC CI was repeated for a total of 20 animals. We chose to repeat the CI in the case of WT:deltamcpC to ensure that the modest phenotype was reproducible and also because early (2 dpi) infection in this model is more variable than later timepoints. This replication was successful.
Randomization	Mice were assigned randomly to cages prior to experimentation. For all of the in vitro experiments, randomization is not relevant because samples were divided into groups based on the genotype of the bacteria, i.e. WT versus mutant.
Blinding	In experiments where the introduction of bias is possible, samples were blinded during data analysis. This includes the RNA seq analysis and all of the microscopy. All other experiments were not blinded because the output was counting of colony forming units which cannot be swayed by bias.

Reporting for specific materials, systems and methods

We require information from authors about some types of materials, experimental systems and methods used in many studies. Here, indicate whether each material, system or method listed is relevant to your study. If you are not sure if a list item applies to your research, read the appropriate section before selecting a response.

Materials & experimental systems

n/a	Involved in the study	
☐	☒	Antibodies
☐	☒	Eukaryotic cell lines
☒	☐	Palaeontology
☐	☒	Animals and other organisms
☐	☒	Human research participants
☒	☐	Clinical data

Methods

n/a	Involved in the study	
☒	☐	ChIP-seq
☐	☒	Flow cytometry
☒	☐	MRI-based neuroimaging

Antibodies

Antibodies used	Goat polyclonal anti-Salmonella CSA-1 (KPL, Cat #01-91-99), Rabbit polyclonal anti-mCherry (Thermo Fisher, Cat #PA5-34974), Mouse monoclonal anti-cytokeratin clone C-51 (Thermo Fisher, Cat #MA1-19035), Donkey anti-rabbit Alexa Fluor 568 (Thermo Fisher, Cat #A10042), Donkey anti-mouse CF633 (Biotium, Cat #20124), Anti-Salmonella H:i antisera (SSI Diagnostica, Cat#23850), Alexa Fluor 488 conjugated goat anti Salmonella CSA1 and Pacific Blue conjugated goat anti Salmonella CSA-1 (made using the Alexa Fluor 488 Antibody labeling kit (Thermo Fisher Cat #A20181 or the Pacific Blue Antibody labeling kit (Cat #P30013) with the above described goat anti-CSA antibody per manufacterer's instructions.
Validation	We used (3) commercially available primary antibodies for immunofluorescence. The first is goat polyclonal anti-Salmonella CSA-1 from KPL. The product datasheet says "This product is designed to react broadly with Salmonella enterica. This includes serotypes of group A, B, C, D, E, and other antigen classes. The intensity of reaction may vary depending on the strain of Salmonella tested. It may also show some cross- reactivity to related Enterobacteriaceae. Product is tested via ELISA techniques as applicable". We use this antibody extensively in our laboratory and have validated that there is no cross reactivity in bovine loops or HeLa cells by using uninfected control tissue and cells. The second primary antibody we used is rabbit polyclonal anti-mCherry from Thermo Fisher, which has been referenced in publications, including McManus et al (DOI: 10.1534/genetics.117.1123). We further validated the specificity antibody by using uninfected control tissue and cells. The third primary antibody we used is mouse monoclonal anti-cytokeratin clone C-51 from Thermo Fisher. The datasheet claims it is reactive against bovine and we validated this in our study by using bovine tissues and seeing the correct pattern of location to epithelial cells.

Eukaryotic cell lines

Policy information about cell lines

Cell line source(s)	HeLa and C2BBe1 (clone of Caco-2) cells were obtained from the American Type Culture Collection Cat #ATCC CCL-2 and ATCC CRL-2102.
Authentication	Cell lines were not authenticated, but were obtained directly from ATCC and used within 15 passages of receipt.
Mycoplasma contamination	Cell lines were not tested for mycoplasma. Cells are used for a limited number of passages (15).
Commonly misidentified lines (See ICLAC register)	No commonly misidentified cell lines were used in this study.

Animals and other organisms

Policy information about studies involving animals; ARRIVE guidelines recommended for reporting animal research

Laboratory animals	Male and female C57BL/6J mice from Jackson were between 6-8 weeks old. The three calves obtained from Texas A&M University Veterninary Medical Park were between 4-6 weeks old (2 males and 1 female). Housing, husbandry and infection details are provided in the methods section.
Wild animals	This study did not involve wild animals.
Field-collected samples	The study did not involve field collected samples.
Ethics oversight	All animal studies were carried out following the recommendations in the Guide for the Care and Use of Laboratory Animals, 8th Edition (National Research Council), and the animal study protocols were approved by the Rocky Mountain Laboratories Animal Care and Use Committee. Protocol numbers 2016-035 and 2019-056. Bovine ligated ileal loop surgeries were approved by the Texas A&M University Institutional Animal Care and Use Committee (2017-0445).

Note that full information on the approval of the study protocol must also be provided in the manuscript.

Human research participants

Policy information about studies involving human research participants

Population characteristics	Human blood donations were collected from volunteers and were completely blinded to our lab.
Recruitment	Human blood donations were collected from volunteers by the Department for Transfusion Medicine, NIH, with signed informed consent, acknowledging that the donation would be used for research by NIH intramural investigators.
Ethics oversight	Blood collection was approved by the NIH Institutional Review Board (Protocol 99-CC-0168). Samples were maintained and used anonymously.

Note that full information on the approval of the study protocol must also be provided in the manuscript.

Flow Cytometry

Plots

Confirm that:

- [x] The axis labels state the marker and fluorochrome used (e.g. CD4-FITC).
- [x] The axis scales are clearly visible. Include numbers along axes only for bottom left plot of group (a 'group' is an analysis of identical markers).
- [x] All plots are contour plots with outliers or pseudocolor plots.
- [x] A numerical value for number of cells or percentage (with statistics) is provided.

Methodology

Sample preparation	Bacteria containing a GFP reporter or control bacteria were fixed in 2.5% paraformaldehyde and stained with Syto41, followed by washing with PBS.
Instrument	A BD LSR II flow cytometer from BD Bioscience was used.
Software	FlowJo Software (Tree Star) was used for analysis.
Cell population abundance	At least 100,000 events were captured per sample. Bacteria were identified using a DNA stain - Syto 41 to ensure purity.

Gating strategy

Syto41 positive gating was drawn using control bacteria that were not stained with Syto41. GFP positive gating was drawn using control bacteria with no GFP.

☒ Tick this box to confirm that a figure exemplifying the gating strategy is provided in the Supplementary Information.
