# Supplementary material for: Regulatory protein HilD stimulates Salmonella Typhimurium invasiveness by promoting smooth swimming via the methyl-accepting chemotaxis protein McpC
Source: Nat Commun. 2021 Jan 13;12:348. doi: 10.1038/s41467-020-20558-6 (PMC7806825; doi:10.1038/s41467-020-20558-6)
Supplement: Supplementary file 2 — Peer Review File [file 41467_2020_20558_MOESM2_ESM.pdf]

Editorial Note: This manuscript has been previously reviewed at another journal that is not operating a transparent peer review scheme. This document only contains reviewer comments and rebuttal letters for versions considered at *Nature Communications*.

REVIEWERS' COMMENTS

Reviewer #1 (Remarks to the Author):

The authors have done a fantastic job of addressing reviewer's concerns. I recommend publication.
